# Long- and Short-Term Selective Forces on Malaria Parasite Genomes

**DOI:** 10.1371/journal.pgen.1001099

**Published:** 2010-09-09

**Authors:** Sanne Nygaard, Alexander Braunstein, Gareth Malsen, Stijn Van Dongen, Paul P. Gardner, Anders Krogh, Thomas D. Otto, Arnab Pain, Matthew Berriman, Jon McAuliffe, Emmanouil T. Dermitzakis, Daniel C. Jeffares

**Affiliations:** 1 Bioinformatics Centre, University of Copenhagen, Copenhagen, Denmark; 2 Biotech Research and Innovation Centre, University of Copenhagen, Copenhagen, Denmark; 3 Center for Social Evolution, University of Copenhagen, Copenhagen, Denmark; 4 Statistics Department, University of Pennsylvania, Philadelphia, Pennsylvania, United States of America; 5 Google, Inc., Mountain View, California, United States of America; 6 Wellcome Trust Sanger Institute, Cambridge, United Kingdom; 7 RNA Genomics, European Bioinformatics Institute, Cambridge, United Kingdom; 8 Computational Bioscience Research Center, King Abdullah University of Science and Technology, Jeddah, Saudi Arabia; 9 Department of Genetic Medicine and Development, University of Geneva, Geneva, Switzerland; 10 Department of Genetics, Evolution and Environment, University College London, United Kingdom; Stanford University, United States of America

## Abstract

Plasmodium parasites, the causal agents of malaria, result in more than 1 million deaths annually. Plasmodium are unicellular eukaryotes with small ∼23 Mb genomes encoding ∼5200 protein-coding genes. The protein-coding genes comprise about half of these genomes. Although evolutionary processes have a significant impact on malaria control, the selective pressures within Plasmodium genomes are poorly understood, particularly in the non-protein-coding portion of the genome. We use evolutionary methods to describe selective processes in both the coding and non-coding regions of these genomes. Based on genome alignments of seven Plasmodium species, we show that protein-coding, intergenic and intronic regions are all subject to purifying selection and we identify 670 conserved non-genic elements. We then use genome-wide polymorphism data from *P. falciparum* to describe short-term selective processes in this species and identify some candidate genes for balancing (diversifying) selection. Our analyses suggest that there are many functional elements in the non-genic regions of these genomes and that adaptive evolution has occurred more frequently in the protein-coding regions of the genome.

## Author Summary

Malaria causes debilitating ill-health in millions of people and kills about one million people annually, mostly young children. It is caused by a single-cell Plasmodium parasite transmitted to humans via mosquito bites. It is difficult to control this parasite because variable genetic make-up enables it to evade detection by vaccines and because drug resistance has repeatedly evolved. Therefore any progress in our understanding of the evolution and genetic variation of the parasite will be central to controlling the parasite. Genic regions that encode proteins are comparatively easy to characterize, whereas non-genic regions are poorly understood. We compare the genomes of seven distantly-related Plasmodium species and find that some of the non-genic regions are very similar between species. The absence of significant evolutionary differences between these non-genic regions implies that they play an important role in the survival of the organism. We then compare the genomes of thirteen different strains of *Plasmodium falciparum*. It is currently accepted that several families of antigenic parasite genes evolve rapidly. However, using two methods we demonstrate that many other genes have also undergone adaptive evolution.

## Introduction

Half of the world's population is at risk of contracting malaria from Plasmodium species [1], so an understanding of their biology has considerable potential to influence human health. An understanding of evolution and natural selection are particularly important, because malaria control is limited by the evolution of resistance to anti-malarial drugs [2]–[5] and high levels of genetic variation in parasite surface proteins, which hinder natural and vaccine-induced immunity [6], [7]. An understanding of selection in these genomes can also contribute to our understanding of their function. For example, intronic and intergenic regions have been shown to be more conserved than neutrally evolving sites [8], [9], suggesting that purifying selection has been acting to conserve functional elements in non-genic regions of the genomes.

In this study we describe selection in Plasmodium genomes over the long term using alignments of the genomes of seven Plasmodium species, showing that there is considerable constraint outside protein-coding regions and identifying 670 non-genic conserved elements. We describe selection in the short term using genome-wide polymorphism data from 13 strains of *P. falciparum*. This analysis suggested that protein-coding exons were more likely to be subject to non-neutral (adaptive) than non-genic regions.

## Results

### Long-term selective forces in seven Plasmodium species

#### Global levels of selective constraints in Plasmodium genomes

We first sought to gain an overview of purifying selection in Plasmodium genomes, because regions of the genome that are subject to purifying selection are likely to contain functional elements. The action of purifying selection can be detected and quantified by estimating the ‘selective constraint’, the proportion of nucleotide substitutions that have been removed by purifying selection [10]. In practice, selective constraint is estimated as the rate of divergence of a region relative to a set of selectively unconstrained sites [11], [12].

**Figure 1:**
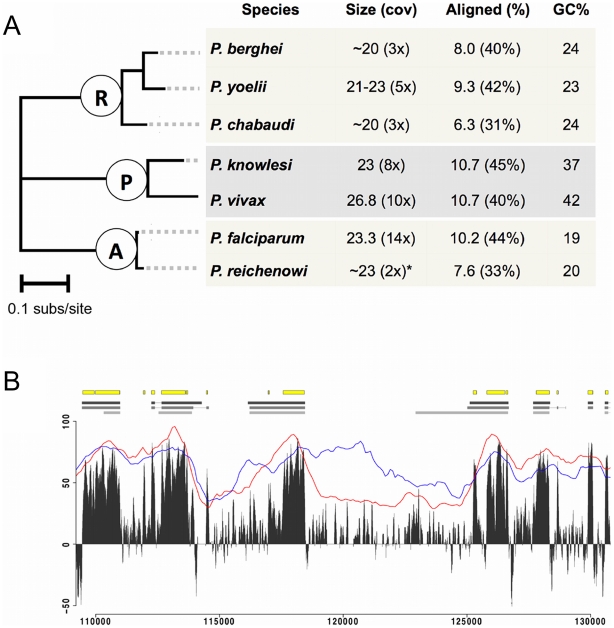
Genome-wide alignment of Plasmodium species. A) Summary of genome data in the context of Plasmodium phylogeny. Branch lengths are generated using the HKY85 model of full alignment, an approximate scale bar of 0.1 substitutions/site is shown. The three main clades are labelled according the mammalian hosts; R (rodent-infecting), P (primate-infecting, human *P. vivax*, human/macaque *P. knowlesi*), A (ape-infecting, human *P. falciparum*, chimpanzee *P. reichenowi*). * The *P. reichenowi* genome was generated from read alignments to *P. falciparum*, we assume a genome of ∼23 Mb. B) Alignment quality in a selected alignment block. At the top of the figure, the yellow boxes show conserved elements (CEs) ≥25 nt predicted by GERP, grey boxes and lines show annotated exons and introns for *P. falciparum*, *P. knowlesi* and *P. yoelii* (upper, middle and lower respectively). Below, the grey peaks shows the GERP score over a sliding window of 25 nt (higher GERP scores indicate stronger evidence for conservation). The red and blue lines indicate the percentage of the window that contains aligned nucleotides (*i.e.*: not gaps) and the percentage identity of the alignment, both over a 1000 nt window.

We used exon-anchored genome alignments of the six available assembled genomes, adding a predicted *P. reichenowi* genome by assuming synteny with *P. falciparum* (data used are summarised in Figure 1A) to estimate divergence in the alignable portions of Plasmodium genomes. To ensure robust results despite differences in GC content (Figure 1), we used both a simple model (HKY85 [13]) and a more parameter-rich non-time reversible model. Each model was optimised on three different selections of the alignment (see Materials and Methods), resulting in a total of six optimised substitution models. We then estimated the divergence in the entire alignment, protein-coding exons, introns, intergenic regions, and four-fold degenerate (FFD) codon positions. FFD sites were the most divergent (Table S1), consistent with the expectation that these sites are relatively free to vary without affecting the protein coding sequence. Accordingly, we use FFD sites as proxy for sites that are selectively unconstrained.

We then estimated constraint within each region as 
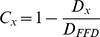



**Figure 2:**
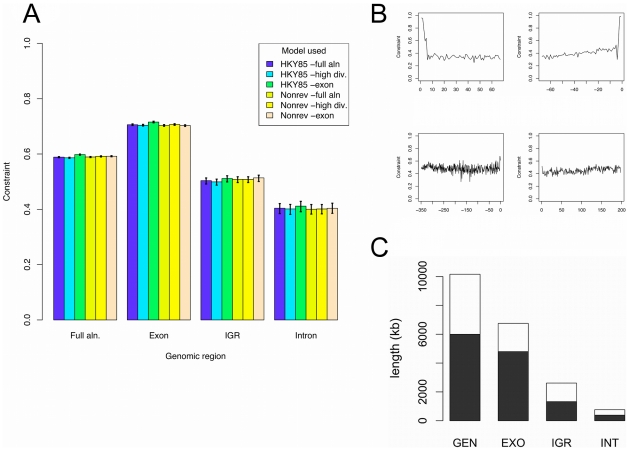
Selective constraint in Plasmodium genomes. A) Selective constraint estimates relative to FFD sites, calculated for the whole alignment (Full aln.), exonic regions, intergenic regions (IGR), and intronic regions using six different models (see text and Materials and Methods for details). Error bars show the minimum and maximum values obtained by bootstrapping, and are enlarged five times to be visible in all cases. Exon regions are the most highly constrained (0.70–0.72), but intergenic regions (0.50–0.51) and introns (0.40–0.41), also show considerable constraint. The genome constraint is 0.59–0.60. B) Selective constraint estimates near intron (upper panels) and intergenic (lower panels) boundaries. Intron (donor) shows the 5′ end of an intron, Intron (acceptor) the 3′ end. Intergenic (promoter and terminator) shows the regions upstream and downstream of protein-coding genes. Annotations of a particular feature (intron, intergenic region) do not always coincide exactly between species, here we show the shortest consensus. Using the longest consensus instead did not affect results significantly (see Figure S5. for a comparison). C) The number of *P. falciparum* nucleotides that are ‘well aligned’ (aligned in ≥four species) and the number that are constrained for the whole genome, exons, intergenic regions and introns.

Where C*_x_* is constraint within a region *x*, D*_x_* is divergence within this region and D*_FFD_* is divergence within four-fold degenerate coding sites. We found that overall constraint of the alignments is 0.59–0.60, and that constraint estimates were consistent across all substitution models (Figure 2A, Table S1). As expected, the most constrained regions were exons (0.70–0.72 constraint), but intergenic regions and introns also showed high levels of constraint, 0.50–0.51 and 0.40–0.41 respectively, indicating that a large fraction of the non-protein coding genome contains functional elements. A similar pattern of constraint was observed with independent constraint estimates within each of the three mains clades (Figure S1). These estimates only apply to the alignable parts of the genomes, so will exclude genes and gene families that are restricted to one species (such as the variant surface antigens *Var*, *Kir etc.*) [14]–[16], and any other highly divergent regions that are not aligned. Differences in alignment between intergenic and exonic regions complicate the interpretation of relative levels of constraint in exons *vs.* other genomic regions. However, by examining how many intergenic, exonic and intronic sites were well aligned (aligned in at least four species) and multiplying this number by the constraint, we estimate that there are approximately ∼4,700 kb constrained exonic sites in the *P. falciparum* genome compared to ∼1,300 kb of constrained intergenic sites (Text S1, Table S2).

Finally, position-by-position analysis of constraint near exon starts and ends showed that intron constraint was strongest within 10 nt of exon boundaries, presumably corresponding to splice motifs, but otherwise intronic and intergenic constraint showed only modest variation with increasing distance from gene boundaries (Figure 2B). This suggests that functional elements are distributed throughout these regions.

#### Conserved Elements in Plasmodium genomes

To assess the functional implications of the constrained sites that reside outside known protein-coding regions, we identified clusters of constrained sites using the genomic evolutionary rate profiling method (GERP) [17]. This algorithm identifies windows of an alignment where the evolutionary rate is significantly slower than the neutral rate. GERP identified 27,575 such conserved elements (CEs), with 10% false discovery rate (FDR). Single nucleotide polymorphism (SNP) data generated from *P. falciparum* (see below) confirmed that these conserved elements were subject to purifying selection within this species, showing that they were not artifacts of ‘mutational cold-spots’ (Text S1). Estimated selective constraints in CE regions were high; 0.78 in exonic CEs, 0.73 intergenic CEs and 0.70 in intronic CEs (Table S3), confirming that some intergenic elements are as conserved as exons.

Since false discovery rates were higher for elements <25 nt long (Figure S2), and short elements proved to contain too little information to characterise *in silico*, all further analysis and discussion was restricted to the 17,949 CEs ≥25 nt long. The majority of these longer CEs (96%) were at least partially exonic (overlapped annotated protein-coding exons in at least one of *P. falciparum*, *P. knowlesi* or *P. yoelii*), 70% overlap exon annotations in all three species (Table 1).

**Table 1:** Conserved Element (CE) annotations.

Annotation used	Exon (%)	Intron (%)	Intergenic (%)
*P. falciparum*	16,420 (91)	5,220 (29)	2,398 (13)
*P. yoelii*	13,468 (75)	4,039 (22)	2,564 (14)
*P. knowlesi*	16,873 (94)	5,488 (30)	2,321 (12)
All three of above	12,743 (70)	3,083 (17)	936 (5.2)
Any one of the above	17,271 (96)	6,413 (35)	4,218 (23)

The number of conserved elements ≥25 nt that overlap various annotations in three Plasmodium species (one from each clade), or combinations of species.

**Figure 3:**
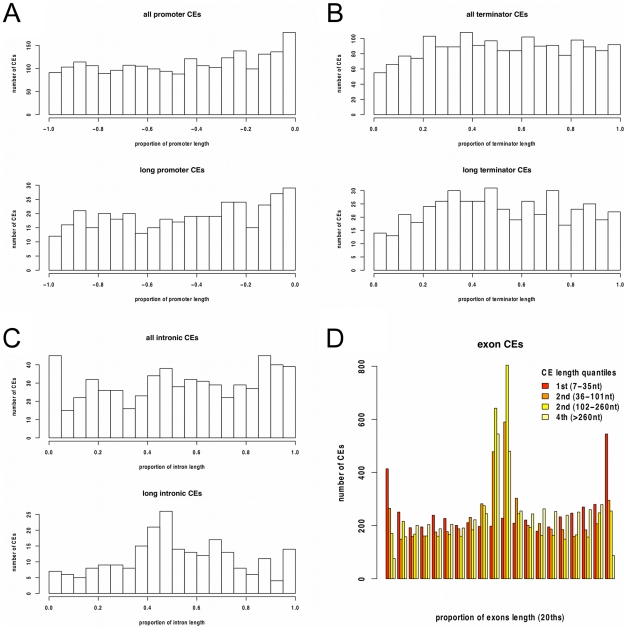
Constrained Element distribution. The distribution of conserved elements (CEs) across the lengths of promoter regions (A), terminator regions (B), introns (C) and exons (D). Positions are the midpoint of the CE with respect to the proportion of the length of the annotated exon, promoter, *etc.*, *e.g.*: a CE at 0.1 is centred at 1/10^th^ the length of the exon. All positions are with respect to the *P. falciparum* annotation. Since UTRs are not well annotated in *P. falciparum*, we divide all intergenic regions (IGR) in half, and refer to any IGR half that is closer to a gene start codon as a promoter region, and any IGR half that is closer to a gene stop codon as a terminator region. Exon CEs are plotted in quartiles because long exon CEs frequently cover most the exon (median exon length is 192 nt), resulting in long exon CE midpoints being preferentially located in the middle of exons.

While the majority of the CEs were protein-coding exons, 670 of these longer non-exonic conserved elements did not overlap exon annotations in either *P. falciparum*, *P. knowlesi* or *P. yoelli*. These CEs were relatively evenly distributed throughout intergenic and intronic regions (Figure 3), consistent with the analysis of selective constraint. This shows that functional elements are equally likely to be located close to, or distant from protein-coding genes.

#### Function of Conserved Elements

**Figure 4:**
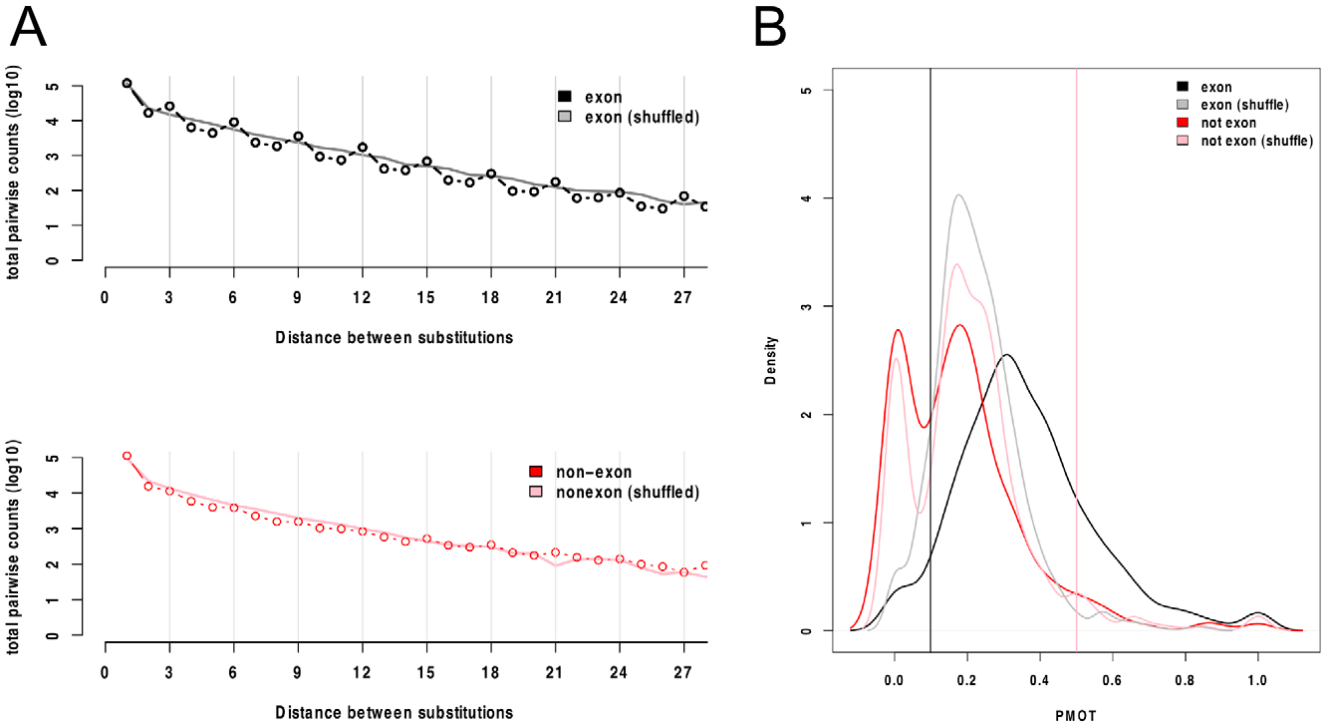
Protein-coding potential in Constrained Elements. A) The total counts of all pairwise comparisons within conserved elements plotted against the distance between substitutions. A bias towards multiple-of-three spaced substitutions (3, 6, 9 *etc.*) is expected in protein-coding elements. Above, counts in 670 exonic CEs (black circles) and these exonic CEs after shuffling alignments (grey line). There is an excess of substitutions separated by a multiples of three nucleotides in the exonic elements compared to the shuffled data. Below, counts in the 670 non-exonic CEs (red circles) and these non-exonic CEs after shuffling alignments (pink line). There is a small excess of multiple-of-three-spaced substitutions, observable at x-axis positions 15, 18 and 21. B) The distribution of the proportion of multiple-of-three-spaced substitutions scores (PMOT) for the exonic CE control set (black lines) and non-exonic CEs (red line). The shuffled exon set (grey line), and shuffled non-exon set (pink line) are also shown. The non-exon set closely resembles the shuffled non-exon set. The vertical black line shows the 5^th^ percentiles of exon PMOT values, elements with PMOT scores less than this are unlikely to be protein-coding. The vertical pink line shows the 95^th^ percentile of shuffled non-exon PMOT values, elements with PMOT values above this are very likely to be exons.

To assess the possibility that some of the 670 non-exonic CEs are un-annotated protein-coding exons, we first examined substitution patterns within CE alignments. Exons are expected to have an excess of substitutions in synonymous sites. Because synonymous sites are usually at third codon positions, this excess can be observed as a bias in substitutions separated by multiples of three nucleotides compared to shuffled alignments (Figure 4A, top). For the non-exonic CEs this bias is very slight (Figure 4A, bottom), consistent with their being largely non-protein-coding. This was confirmed by comparing the proportion of multiple-of-three-spaced substitutions between non-exonic CEs, length-matched exonic controls, and shuffled alignments (Figure 4B).

Further analysis of the 670 non-exonic conserved elements was consistent with only a minority being un-annotated exons. First, analysis of the similarity of CEs to the genomes of related Alveolates using translated blast searches (tblastx) showed that non-exonic CEs had significantly lower blast bit scores than exonic CEs (Text S1, Table S4). Secondly, analysis of expression levels using RNA-Seq data from a recent study of *P. falciparum* [18], suggested that these elements were not more highly expressed than expected of intronic or intergenic regions of the genome. Finally, manual scrutiny of 20 non-exonic elements in the context of the *P. falciparum* DNA sequence, gene annotation and RNA-Seq data suggested that only 25% were possible protein-coding exons (see Text S1 for details of both analyses).

Since only a few of the non-exonic CEs appear to encode proteins, another possibility is that they encode structured, non-protein-coding RNAs (ncRNAs). Only 239 of the 16,649 CEs in *P. falciparum* (1.4%) overlap previously discovered ncRNAs [19], [20], including exonic antisense ncRNAs. However it is possible that there are many more, as yet undiscovered ncRNAs in these genomes. To investigate this we scored each CE with the structural RNA predictor RNAz [21], controlling for false positives by comparing to scores for simulated alignments, generated using SISSIz [22]. The RNAz algorithm predicted significantly higher support vector probabilities (the probability that an element is a ncRNA) for both exonic CEs and non-exonic CEs than for the simulated alignments (Mann-Whitney tests, P<2.2×10^−16^ and P = 1.70×10^−7^), indicating that some encode genuine structural RNAs. Using the conservative threshold of RNAz support vector probability >0.95, and taking the false discovery rate into account, we estimate that 644 of the exonic and 22 non-exonic CEs encode a structured ncRNA (more detail is provided in Text S1). Since only 239 of our CEs overlap known ncRNAs, this suggests that there are many more structured RNAs that remain to be discovered, particularly within protein-coding exons.

### Short-term selective forces in *P. falciparum*

The analysis of the divergence between distantly related species facilitates analysis of purifying selection (constraint). However, there are several types of selection acting simultaneously on genomes that are less well described from this data, particularly in non-genic regions that are difficult to align between highly divergent species. Firstly, adaptive (or directional) evolution, which acts to change the nucleotide sequence from its ancestral state [23]. There is also evidence that a few genes are subject to balancing selection, which acts to maintain multiple alleles (different versions of a gene) in a population [24]. Frequency-dependent balancing selection, where rare alleles have a selective advantage, is thought to be particularly important in Plasmodium genes encoding surface-exposed proteins that are targets of acquired immunity [25]–[29].

To examine these types of selection, we generated genome-wide genetic diversity (polymorphism) data from thirteen *P. falciparum* isolates (strains) and the divergence between *P. falciparum* and the chimpanzee parasite *P. reichenowi*, using publicly available ABI capillary reads (Table S5, NCBI dbSNP accessions: ss# 159747249–159815961). We called 69,805 SNPs within *P. falciparum* and 190,631 fixed differences between *P. falciparum* and *P. reichenowi*. We were able to obtain a coarse minor allele frequency (MAF) estimate for 54,641 SNPs and a derived allele frequency estimate for 24,573 SNPs (see Materials and Methods). See Table S6 for further summary statistics.

#### Purifying selection is consistent in the medium and short term

We expect the degree of purifying selection acting on most genes to be relatively consistent over evolutionary time. The synonymous/non-synonymous rate ratio (dN/dS) provides an estimate of purifying selection between two species [23], and has been calculated for *P. reichenowi - P. falciparum* [9], which are thought have diverged approximately 6 million years ago [30]. Since these two species diverged relatively recently, this measure gives an indication of constraint over the medium term. As an estimate of the constraint over the short term within *P. falciparum*, we calculated the median derived allele frequency (DAF) for each gene with at least fifteen DAF calls, and compared these to the corresponding dN/dS values. Genes that are subject to strong purifying selection will be expected to have lower median DAF and lower dN/dS. As expected, the median DAF and dN/dS showed a weak but significant correlation (Spearman rank P = 2.03×10^−4^, *r* = 0.16), confirming that constraint is generally consistent. We also found that the extent of purifying selection within *P. falciparum* (using DAF) was weakly correlated with long term constraint (from the seven-species alignment) in exonic and intergenic regions (Text S1).

#### Recent selection within the *P. falciparum* genome

Our analysis of divergence between species showed that exons are subject to more extensive purifying selection than introns or intergenic regions. Given this, we expect to observe the same selection within species. Purifying selection within species can be detected by an excess of rare alleles, relative to some control [23]. Consistent with this expectation, we observed a significant shift to low minor allele frequencies (MAF) in *P. falciparum* exons relative to intergenic regions (one-sided Mann-Whitney test P = 1.4×10^−4^). No other comparisons of allele frequencies between exons, introns or intergenic regions were significant (Table S7).

We would also expect greater purifying selection acting on non-synonymous sites, relative to four-fold degenerate (FFD) sites, because the latter do not alter the protein sequence and will therefore contribute less to the phenotypes that are subject to purifying selection. However, this is not what we observe. Non-synonymous SNPs show an excess of *higher* derived allele frequencies (non-synonymous vs. FFD, DAF P = 7.7×10^−3^, MAF P = 0.065 (ns)). The fact that this pattern is significant in the genome-wide test suggests that many non-synonymous sites have been subject to adaptive or balancing selection.

Such non-neutral (adaptive or balancing) selection may be due to selection to evade the human immune response. Three gene families, *Var*, *Rifin* and *Stevor*, encode parasite proteins that are believed to dominate the human immune response to *P. falciparum* [14]–[16]. Consistent with this expectation, we find that exonic sites in *Var*, *Rifin* and *Stevor* genes have a significant excess of high frequency alleles compared to intergenic sites (Var gene exon MAF vs. Var gene intergenic MAF, P = 7.38×10^−10^, see Table S7). This is in contrast to the genome as a whole; genome-wide, exon MAF is significantly *less* than intergenic MAF. As a result, it is possible that these genes are responsible for the unexpected excess of non-synonymous SNPs. However, the excess of high frequency derived alleles (DAF) in non-synonymous sites compared to four-fold degenerate sites remains significant after removing these genes from our analysis (P = 3.1×10^−3^), suggesting that many other genes in the genome are subject to adaptive or balancing selection.

To analyse this further we examined allele frequencies using a GO-Slim gene ontology categorization of genes consisting of 23 broad biological categories [9]. We compared allele frequency estimates (MAF and DAF) for all SNPs within each GO-Slim category to all other SNPs. Categories that showed an excess of rare alleles (indicating strong purifying selection) were nucleic acid metabolism, regulation of cellular physiological process, amino acid and derivative metabolism, cell organization and biogenesis and protein metabolism. In contrast, SNPs in cell communication, adhesion to host, and avoidance of host defenses (which all contain overlapping sets of genes) showed an excess of high minor allele frequencies consistent with less constraint and/or adaptive or balancing selection (see Table S8). This GO analysis is consistent with a previous analysis using dN/dS [9].

#### Signals of adaptive or balancing selection

To investigate adaptive (directional) selection further, we examined whether adaptive evolution between *P. falciparum* and the chimpanzee parasite *P. reichenowi* had occurred predominantly in exons. We used the McDonald-Kreitman test [31] which can quantify the proportion of substitutions (α) that have been fixed due to adaptive evolution in coding sites and non-coding sites [32], [33]. We estimate that α = 0.41 in non-synonymous sites (95% confidence interval 0.39–0.45), 0.10 in intergenic regions (0.04–0.15), and 0.11 in introns (−0.07–0.28). Results were the same when we excluded *Var*, *Rifin* and *Stevor* genes. To obtain an estimate of the number of adaptive changes, we multiply McDonald-Kreitman test α estimates by the total number of fixed differences between *P. falciparum* and *P. reichenowi*. We observe 77,275 fixed non-synonymous differences in exons, hence our estimate is that 30,910 adaptive chances have occurred (77,275×0.4). Similarly, we estimate that 3,517 adaptive changes have occurred in intergenic regions and 1,102 in introns (35173×0.1 and 11025×0.1 respectively). Hence, we estimate that the majority of the adaptive substitution events have occurred in the protein-coding exons of *P. falciparum*.

#### Candidate genes subject to balancing selection

There is considerable interest in identifying Plasmodium genes that show signs of balancing selection, because such genes may be the targets of the human acquired immune response and therefore be vaccine candidates [34]. To investigate which genes are the most likely to be subject to balancing selection, we used two independent tests of the data, Tajima's D and the neutrality index (NI) from the McDonald-Kreitman test, both of which are thought to be suitable tests for balancing selection [35], [36]. Since these are orthogonal tests for balancing selection (they use independent properties of the data), genes that have high ranks in both these statistics are good candidates for balancing selection. There were 591 genes for which we can calculate NI and D. Importantly, because of the stringency of the read alignment method that we used to identify SNPs, the variant surface antigen (*Var*) genes are generally not well covered by this analysis.

**Figure 5:**
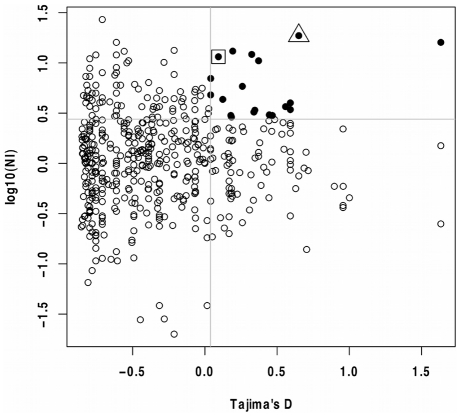
Tajima's D and MK test NI values for *P. falciparum* genes. Scatter plot of the McDonald-Kreitman test neutrality index (NI) and Tajima's D. Grey lines show the 80^th^ percentiles of NI and D. Filled circles show the 19 genes that have both NI and D values greater the 80^th^ percentiles. The square box indicates *AMA1* (PF11_0344) and the triangle indicates *RhopH3* (PFI0265c).

To establish a list of suitable candidates, we selected those genes that have both NI values greater the 80^th^ percentile of NI and D greater the 80^th^ percentile of D values (19 genes, Table 2, Figure 5). The fact that there are less genes above both 80^th^ percentiles than we expect by chance (expect 0.2×0.2×591 = 24), suggests that diversifying selection is not common in *P. falciparum*. This candidate list includes *AMA1* which is highly polymorphic in many populations due to diversifying selection and currently a leading vaccine candidate [25], [37], [38]. *Rhoph3*, a rhoptry protein triggering immune responses in patients from endemic regions is also in our candidate list [39], as are several candidates with functions suggestive of host-parasite interactions. This suggests that this set of genes is enriched for genes that are subject to diversifying selection. Mu *et al.* [29] also defined a candidate list of highly polymorphic genes that they suggest will be potential immune or drug targets, two genes are present in both lists (AMA1, and MAL7P1.66, a mitochondrial ribosomal protein S5 precursor).

**Table 2:** Candidate genes for balancing selection in *P. falciparum*.

Systematic Name	NI	log10(NI)	D	Annotation (Gene)
MAL7P1.228	3.4	0.5	0.592	Heat shock protein 70, pseudogene (PfHSP70-x)
MAL7P1.229	3.0	0.5	0.446	Cytoadherence linked asexual protein
MAL7P1.27	3.7	0.6	0.558	Chloroquine resistance transporter (CRT)
MAL7P1.66*	3.4	0.5	0.346	Mitochondrial ribosomal protein S5 precursor
MAL7P1.89	13.1	1.1	0.193	Dynein heavy chain
MAL8P1.150	12.1	1.1	0.324	Adenylyl cyclase beta (ACbeta)
PF07_0047	2.9	0.5	0.184	AAA family ATPase, CDC48 subfamily (Cdc48)
PF07_0066	3.0	0.5	0.180	Conserved Plasmodium protein, unknown function
PF10_0295	4.0	0.6	0.592	Conserved Plasmodium protein, unknown function
PF10_0366	10.5	1.0	0.372	ADP/ATP transporter on adenylate translocase
PF11_0344*	11.5	1.1	0.094	Apical membrane antigen 1 (AMA1)
PFB0405w	5.8	0.8	0.260	6-cysteine protein/transmission-blocking target antigen (P230)
PFC0640w	3.2	0.5	0.339	CSP and TRAP-related protein (CTRP)
PFE0340c	4.8	0.7	0.040	Rhomboid protease (ROM4)
PFE0465c	7.0	0.8	0.041	RNA polymerase I
PFF1345w	3.0	0.5	0.467	Transportin
PFI0170w	4.3	0.6	0.124	Conserved Plasmodium protein, unknown function
PFI0265c	18.7	1.3	0.650	High molecular weight rhoptry protein 3 (RhopH3)
PFI1260c	16.0	1.2	1.633	Histone deacetylase (HDAC1)

*Also predicted to be highly polymorphic genes that are potential immune or drug targets in Mu *et al.* [29].

## Discussion

While molecular assays identify considerable activity (transcription, methylation, transcription-factor binding) in the non-genic regions of eukaryote genomes, it is not always clear to which extent these are functional. For example, a principle finding of the ENCODE project was that many ‘active’ genome regions (transcribed, or bound by proteins) were not conserved between species [40]. Studies of selective constraint in eukaryote genomes without reference to the ‘activity’ of the genomic region, though not without their caveats, provide an alternative view of which genomic regions are most important for the function of the genome. Intergenic constraints of 0.05–0.15 have been estimated in the human genome [41], [42], between 0.03 and 0.49 in Drosophila (depending on the method) [10], [33], and 0.18 in *Caenorhabditis* nematodes [43]. From a recent study of divergence between *Saccharomyces* yeasts [44], we can obtain an estimate of 0.53 for constraint in budding yeast (Constraint = 1−(intergenic divergence/synonymous divergence)). Our estimate of constraint in intergenic regions (0.5) is similar to yeast. This conclusion is consistent with the variety of putative non-genic functional elements have been predicted in Plasmodium genomes [45]–[51]. All these analyses illustrate that there is much to be discovered about the function of even small eukaryote genomes.

We suspect that the current analysis has detected only a minority of the non-genic conserved elements in these genomes. While 2,611 kb of the *P. falciparum* genome is aligned to at least three other species, the total length of all the intergenic conserved elements we identified was only 25 kb. This is probably due to methodological limitations as well as to biological factors. Since our alignment was exon-anchored, it is possible that conserved elements that are distant from exons will not be well aligned. While 42% of exon-annotated columns are well aligned (aligned with 4 species), this only holds for 24% of intergenic-annotated columns (Figure S3). Some functional intergenic elements, such as transcription factor binding sites, will be inherently difficult to discover with GERP, or any alignment-based method [17], because they are short and may not be positionally conserved [52].

Both divergence data from Plasmodium species and the excess of rare minor alleles (MAF) in exons indicated that exons are subject to more constraint than introns or intergenic regions. However, consistent with a previous study [53], our data shows an excess of high frequency derived alleles in non-synonymous sites compared to four-fold degenerate sites. Since our allele frequency estimates are derived from a sample of parasites collected from several populations (Table S5), this could be due either to balancing, or else to differential directional selection between populations. Since there is no significant correlation between the by-gene estimates of the McDonald Kreitman test neutrality index and D (Spearman rank P = 0.31, r = 0.04), the high D values do not appear to be generally caused by balancing selection. The more likely explanation is that there are differential selective pressures between the populations. This is analogous to differences in non-synonymous allele frequencies in African and European human populations, where the explanation is that populations that have smaller ancestral population sizes have reduced efficacy of purifying selection [54]. Consistent with this, Neafsey *et al.* [53] found that non-synonymous sites are more differentiated (have higher F_ST_) between continents than synonymous sites.

Our estimates of the proportion of substitutions fixed by adaptive evolution (a, exons 0.4, introns 0.1, intergenic 0.09) is consistent with exons being the most frequent genomic location of positive selection. These estimates are broadly similar to the estimates for Drosophila in which exon, intron, intergenic estimates were 0.45, 0.19, 0.15 respectively [33], though estimates vary depending on the method [55]. However, it has been shown that artifactual estimates of α can be generated when there has been an increase in population size and weakly deleterious synonymous mutations have been fixed [56]. Both the existence of weakly deleterious synonymous codons [57] and an increase in population size [58], [59] are likely for *P. falciparum*, so we should regard the absolute value of our estimates with caution.

### Conclusion

In summary, we show that there is considerable constraint in intergenic regions and introns. We identify 670 conserved non-genic elements and our analysis suggest that only a minority of these are un-annotated protein-coding exons, or structured ncRNAs. We suspect that many more functional non-genic elements remain undiscovered. Our analysis is consistent with the majority of non-neutral (directional or balancing) selection events having occurred in *P. falciparum* exons. Genetic diversity data collected from within populations and divergence data from more closely related Plasmodium species, both of which will soon be available, will be required to confirm this prediction.

## Materials and Methods

### Genome data

The following genome versions were used for the alignment:

*P. falciparum* version 2.1.4, July 2007, from ftp://ftp.sanger.ac.uk/pub/pathogens/Plasmodium/falciparum/3D7/3D7.version2.1.4

*Plasmodium knowlesi* version PK4, October 2007, from ftp://ftp.sanger.ac.uk/pub/pathogens/P_knowlesi/Archive/PK4.annotation/

*Plasmodium vivax* is as published in Carlton *et al.* [60], with ordered and orientated contigs in pseudo-chromosomes. Because many of the subtelomeric contigs could not be assigned to the pseudo-chromosomes, they are not present. However, these contigs are extremely AT-rich and contain mainly *Vir* genes, and so they do not align with chromosome regions of other Plasmodium species. Pseudo-chromosomes and annotation are available upon request.

*Plasmodium berghei*, obtained April 2007, from ftp://ftp.sanger.ac.uk/pub/pathogens/Plasmodium/berghei/

*Plasmodium chabaudi*, obtained April 2007, from

ftp://ftp.sanger.ac.uk/pub/pathogens/Plasmodium/chabaudi/

*Plasmodium yoelii*, PlasmoDB version 5.4, from

http://www.plasmodb.org/common/downloads/release-5.4/Pyoelii/

*Plasmodium reichenowi*

A predicted *Plasmodium reichenowi* genome was created by aligning 78,442 *P. reichenowi* ABI capillary reads to the *P. falciparum* genome with SSAHA2, as described previously [9]. Fixed differences were discovered with a minimum phred score of 25. Deletions in the *P. reichenowi* sequence were identified, requiring at least two read alignments identifying an identical deletion before we accepted it. Insertions in *P. reichenowi* could not be included without manipulating the overall alignment. We then assumed that *P. reichenowi* was identical to *P. falciparum* except in locations of fixed differences or deletions in the *P. reichenowi* sequence. We excluded regions of the genome that were non-unique (as described below for SNPs) or lacked read coverage.

### Alignments

The six assembled genomes were repeat masked using Repeat Masker Open-3.0, from http://www.repeatmasker.org, then aligned in two phases. The first phase was synteny mapping. Sub-sequences of the genomes were grouped into syntenic blocks. Each of the six genomes contributed at most one subsequence to a given block, and each block contained sequence from at least two species. Then, in the second phase, a nucleotide-level multiple alignment was constructed within each block.

The synteny map was generated using Mercator [61]. Mercator requires anchor sequences along each genome. In addition, for each anchor, one must specify which anchors on other genomes are strongly similar. We chose as anchors the known and predicted exons in each genome, with the annotations obtained as described. Two anchors, each from a different genome, were deemed similar if the BLAT [62] score of the pair was below 1×10^−50^. The BLAT scores were computed in protein space. Mercator used the anchors and BLAT-similar pairs in a modified k-way reciprocal best hit algorithm [63]. The non-draft genome sequences (*P. falciparum*, *P. knowlesi*, and *P. vivax*) served as scaffolds for the draft species. The result was 170 syntenic blocks. The largest blocks covered parts of all six species and contained entire chromosomes from some of them, while the smallest contained small fragments of just two species. Mercator also produced alignment constraints for each block.

A nucleotide-level multiple alignment within each block was generated with MAVID [64], using the alignment constraints as well as a phylogenetic tree relating the six species. Branch lengths for the tree were estimated with PAML [65], fixing the known topology for these Plasmodium species. Working upwards from the leaves, MAVID associates to each branch node a maximum-likelihood alignment of the sequences in the subtree rooted at that node. The alignment at the root of the full tree is a multiple alignment of all the input sequences. The accuracy and coverage of both the syntenic map and the block alignments were validated manually according to various descriptive statistics.

The predicted *P. reichenowi* genome was then added to the alignment assuming complete synteny with *P. falciparum*.

### Divergence and selective constraint

Alignment sites corresponding to exon, intron, and intergenic categories were extracted based on the annotation for *P. falciparum*, *P. knowlesi* and *P. yoelii* (one species from each of the three main clades), requiring identical annotation in all three species. For calculations of constraint we used two different models: The HKY85 model [13], with transition and transversion rates estimated globally for the whole tree, and a non-time reversible 12 parameter model (Nonrev), with all substitution parameters estimated individually for each of the three main branches in the tree (for a total of 36 substitution rates). To further ensure robustness we estimated the model parameters from three different data sets: A) The full alignment (representing overall selective pressures), B) introns, intergenic, and FFD sites (high divergence and AT content), and C) exons (lower divergence and AT content). Model parameters and branch lengths for the topology shown in Figure 1A were estimated with the maximum likelihood based package Hyphy [66].

The resulting six parameterized models were then used to estimate branch lengths based on the full alignment. Branch lengths for the different genomic regions (exon, intergenic, intron, FFD) were then estimated with each model as scalings of the full alignment tree (the relative divergence over all branches of the tree), by keeping the relative branch lengths within the tree fixed. Relative constraint for each region was calculated as 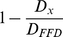, as described in the text.

Variance of the constraint estimates was evaluated by 200 bootstrap replicates of alignment columns, with replacement. This variance is shown as error bars in Figure 2A, for each of the six models, and for each of the four categories (full alignment, exon, intergenic, intron). This measures the statistical variation of the constraint estimates, but does not address the biological variation. The latter is difficult to assess in a meaningful way, due to variation in neutral rate across the genome (Figure S4). The precision of estimates of neutral rate in specific regions of the alignment is limited by the relative scarcity of FFD sites, meaning that for smaller, biologically relevant window sizes, *e.g.* 20 kb, the neutral rate estimates will be highly variant (or non-existent) due to small FFD sample size, leading to uninformative constraint estimates.

To estimate the constraint near gene and exon boundaries, we extracted alignment columns upstream of start codons (termed promoters), downstream of stop codons (terminators) and between exons (introns). In each case, we extracted a region corresponding to one third of the observed median length of the given feature. Introns were then further divided into donors (5′) and acceptors (3′).

To reduce the effect of mis-annotations, we required all species to be present in the alignment, and identical, overlapping annotation features in all the six species for which annotation exists. When the exact boundaries for a feature on the alignment varied in different species' annotation, we chose the maximum start and minimum stop positions (shortest consensus). Results were very similar when we used the longest consensus (minimum start, maximum stop), a comparison can be seen in Figure S5.

To examine whether divergence estimates from Plasmodium species alignments was negatively correlated with DAF, we located each SNP position in the alignment of Plasmodium species. We then calculated the median number of rejected substitutions estimated by GERP in both the 5nt and 11nt window around the SNP.

Estimates of constraint within each main clade were calculated in three ways: First by the same procedure as described above for the full tree, but with each of the three main clades (Rodent, Primate, Ape) scaling independently. Second by estimating the models independently for each of these clades, excluding the long branches to the root. These clade-specific models were then used to estimate the sum of branch lengths (again excluding the long branches) for each of the categories (exon, intron, intergenic, FFD). Both these measures were calculated using regions that were consistently annotated (as exon, intron *etc.*) in P. falciparum, P. yoelii and P. knowlesi. Constraint was then calculated as described above. Third, we calculated constraint within clades as above using regions of the genome that were consistently annotated in all species within the clade (P. knowlesi and P. vivax for the primate clade, P. yoelii, P. berghei and P. chabaudi for the rodent clade, and only P. falciparum for the ape clade).

### Identifying Conserved Elements (CEs)

CEs were identified with *gerpcol* and *gerpelem* programs of GERP [17], version 2.1b (from http://mendel.stanford.edu/sidowlab/downloads/GERP/index.html). Parameters for running *gerpcol* were estimated using Hyphy using a HKY85 model, fitted to the full alignment from FFD sites. These parameters were; a neutral rate of 3.067, and transition/transversion rate of 2.63, and the following phylogenetic tree (((PlaBer:0.0839783,PlaYoe:0.127063):0.126016,PlaCha:0.147872):0.59425, (PlaViv:0.295231,PlaKno:0.213155):0.74449,(PlaFal:0.0141867,PlaRei:0.0420219):0.678922);. The *gerpelem* program of GERP was run using 10% false discovery rate and default parameters.

### Characterising CEs

The average proportion of substitutions that were separated by a multiple of 3 bases (PMOT) was calculated by comparing each sequence in the CE alignment slice to each other sequence. In each pairwise comparison we record the distance between each substitution, and then the proportion of these distances that were multiples of 3. The PMOT score for a CE is the average of these proportions from all pairwise comparisons.

We determined the average tBLASTx bit score for each CE as follows. These BLAST searches are designed to detect protein-coding exons, and the median exon length for *P. falciparum* is 192 nt. So for each Plasmodium species represent in the CE alignment slice, we extract 192 nt of sequence extending out from the midpoint of the CE. We then used this 192 nt sequence to search the Alveolate genomes (Table S4) with NCBI tBLASTx (searched translated nucleotide databases using a translated nucleotide query), accepting only the best hit (irrespective of E-value). For each CE, we record the average bit score for each Plasmodium species represented in the alignment slice. Because all query sequences are the same length bit scores should be comparable between CEs.

CEs were scored for RNA structure using RNAz version 1.0 (http://www.tbi.univie.ac.at/~wash/RNAz) [21]. Since some CEs were longer than the maximum length that RNAz can process, alignments were pre-processed with rnazWindow.pl, (default parameters except no reference sequence), before running RNAz. RNAz was run with default parameters, except predictions were done for both strands). Since rnazWindow.pl has a minimum length cut-off of 50 nt, predictions were not produced for the smaller elements. To give an estimate of the false discovery rate in our data, one simulated alignment was produced for each long CE with SISSIz (Version 0.1) [22]. There were 8726 long exonic CEs where we could produce an RNAz prediction from both the native and the simulated alignment. From these, 98 of the SISSIz alignments had a support vector probability from RNAz >0.95, and 527 of the native alignments had a SV probability >0.95. So false discovery rate (FDR) is 98/527 = 0.19. Similarly non-exonic FDR is 13/33 = 0.4.

Control elements for RNASeq expression analysis were chosen to match the length and GC content of each intergenic CE, but otherwise at random. This produced a set of 406 intergenic ‘non-conserved’ element controls whose length and GC content distributions did not differ significantly from a set of 489 *P. falciparum* intergenic CE elements (Mann-Whitney tests P>0.1). We located a set of 120 intronic ‘non-conserved’ element controls in the same way to compare to the 80 intronic CEs in *P. falciparum*. There was no significant difference between the RNAseq expression levels of intergenic controls and intergenic CE elements, or between intronic controls and intronic CE elements (Mann-Whitney tests, both P>0.05).

### Read alignment and SNP calling

Reads from 13 *P. falciparum* isolates and *P. reichenowi* were used to identify single nucleotide polymorphisms (SNPs) and fixed differences, as follows. See Table S5 for detailed information on SNP calls.

All non-WTSI derived reads were downloaded from the NCBI Trace Archive ftp server (ftp.ncbi.nih.gov/pub/TraceDB/plasmodium_falciparum). Fastq files were mapped to the 3D7 version 2.1.4 reference sequence (ftp://ftp.sanger.ac.uk/pub/pathogens/Plasmodium/falciparum/3D7/3D7.version2.1.4/) using SSAHA2, with the parameters (-seeds 15, -score 250, -tags 1, -diff 15, -output cigar, -memory 300, -cut 5000) and ssaha_pileup code. Ssaha2, ssaha_pileup and associated documentation are available from http://www.sanger.ac.uk/Software/analysis/SSAHA2/.

We excluded non-unique and tandem repeat regions of the genome. Non-unique regions were identified using the SSAHA2 read mapping score (mapping scores range from 0–50). We calculated the mean mapping score for each 2kb window of the genome (with a 100 nt step), using all reads aligned from all isolates (*S_mean_*). The 10^th^ percentile of all *S_mean_* values was 49.5 (*S_mean_* values were frequently 50). We excluded any region within 2kb of a window with *S_mean_* <49.5. We identified tandem repeats using the Emboss application *etandem* (flags -minrepeat 2 -maxrepeat 10), and excluded all tandem repeats with ≥70% identity (to the other repeat). Excluding non-unique regions and tandem repeats left a remainder of 19,664,344 nt of unique non-repeat sequence, 84% of the genome.

As an aid to determining suitable thresholds at which to accept SNP calls, we identified a set of 788 ‘reliable SNPs’ that were called in ≥6 isolates (of the 13 that we examined). Since it is unlikely that we call a false positive 6 times in an identical position, with an identical base, this set of SNPs is enriched for true calls.

We then examined these reliable SNPs in each isolate (*i*) in turn. Each isolate will call a subset of these SNPs. Each SNP call may be from one or more reads, we determine the average phred score (for the SNP base) from all reads, *P_SNP_* ( = total phred score/number of reads calling this base) for each reliable SNP in isolate *i*. We then examine the distribution of reliable SNP *P_SNP_* scores for isolate *i*, comparing it to the distribution of *P_SNP_* scores from all other SNPs called in isolate *i*. The distribution of *P_SNP_* scores from reliable SNPs were always significantly higher than the distribution of *P_SNP_* scores from all non-reliable SNPs (all other SNPs) due to more true calls in the reliable SNP set. Assuming that 95% of reliable calls are correct, we set the minimum phred scores required to call a SNP in isolate *i* as the 5^th^ percentile of the distribution of reliable SNP *P_SNP_* scores. We refer to this value as min(*P_SNP_*, *i*).

At each site in the genome where ssaha_pileup calls a SNP (in any isolate) we accept all SNP or reference base calls from each isolates that is supported by ≥2 reads with a *P_SNP_* ≥min(*P_SNP_*, *i*). When two alleles satisfied these criteria in an isolate, we accept both alleles, since some samples may contain >1 clone. In practice all min(*P_SNP_*, *i*) scores were ∼25, so SNPs are supported by at least 2 reads with cumulative phred ≥∼50. This method identifies reliable SNPs without a bias towards common SNPs. Fixed differences in *P. reichenowi* were accepted if supported by ≥2 reads with an average phred score for an allele ≥25.

Derived allele frequencies were calculated from polymorphic sites with a *P. reichenowi* base call and ≥three isolate base calls, minor allele frequencies from polymorphic sites with ≥four isolate base calls (24,573 DAF calls, 54,641 MAF calls). For gene-specific analysis SNPs were assigned to a gene if they lay within the exons, introns or the half of adjacent intergenic region closer to the gene.

We estimate that the false discovery rate for SNP calling is 1–2%, as follows. We aligned 30,840 reference 3D7 isolate reads from chromosome 12 onto the assembled 3D7 genome and called SNPs as above. Using the thresholds and filters we described above (including only unique regions and excluding tandem repeats), we accept 47 SNPs from 3D7 in chromosome 12 (Table S5). With a similar number of aligned reads, we accept 2,160 SNPs from the PFCLIN isolate and 2,583 SNPs from the IT isolate. If we assume that 47 of the 2160 SNPs from the PFCLIN isolate are false discoveries, then the false discovery rate (FDR) is 47/2160 = 2.1%. With the same reasoning the IT isolate has 1.8% FDR (Table S5). This is probably an overestimate of the FDR because a) some of the 3D7 calls may be correct (*i.e.*: errors in the reference sequence), and b) 3D7 reads are calling SNPs from a larger proportion of chromosome 12 (95% coverage at ≥2 read depth *vs.* ∼75% for IT and PFCLIN isolates, see Table S5).

We also estimated the error rate of SNP allele calling using some Illumina data that was available for the IT and PFCLIN isolates (D. Jeffares, unpublished data). Briefly, 90 genes were chosen from primarily polymorphic but unique regions of the genome, and PCR-amplified from various isolates including PFCLIN and IT. Amplicons were sequenced to high depth with Illumina technology, and mapped to the same reference genome with MAQ. We examined how many of the PFCLIN and IT calls from the SSAHA2-mapped ABI capillary reads matched the alleles called (this study) matched those from the MAQ-mapped Illumina reads, using only sites covered by either 10 or 20 Illumina reads. Differences, which may be false SNP calls in either data set, were of the order of 1–2%, as predicted above. In general, error rates in different regions of the genome (exon, intron, intergenic, FFD, non-synonymous sites) were not significantly different. The exception was that for both isolates intergenic sites had significantly higher error rates than exonic regions (see Table S5). We expect this to result in an artifactual shift of intergenic sites to a lower allele frequencies, because artifactual alleles will be rare. We take this into account by comparing only intergenic *vs.* other intergenic sites. For the comparison of DAF/MAF this would be expected to *diminish* any affect of lower MAF distribution in exons *vs.* intergenic sites. Intronic, exonic, FFD and non-synonymous sites did not differ in error rates.

### Genetic diversity and selection in the *P. falciparum* genome

For each isolate (*i*), a predicted genome was created, for each site in the genome we accept all SNP or reference base calls that were supported by ≥2 reads with a *P_SNP_* ≥min(*P_SNP_*, *i*). Sites without sufficient quantity coverage were denoted ‘N’ and not used in the analysis.

Tajima's D was calculated using Variscan (Version 2.0, [67]), using a fixed number of alleles (4) for each SNP (Variscan chooses a random selection if >4 are available at a site), using only polymorphic sites (Variscan parameters FixNum = 1, NumNuc = 4, UseMuts = 0).

The McDonald-Kreitman test neutrality index was calculated as NI = (Pn/Ps)/(Dn/Ds), where Pn and Ps are non-synonymous and synonymous SNPs and Dn and Ds are non-synonymous and synonymous fixed differences (between *P. reichenowi* and *P. falciparum*).

### Proportion of substitutions fixed by adaptive evolution

It has been shown that the McDonald-Kreitman test can be used to estimate the average proportion of non-synonymous substitutions (α) that have been fixed by adaptive evolution [32], according to the formula 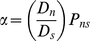, where D*_s_* and D*_n_* are the average number of synonymous and non-synonymous substitutions per gene and P*_ns_* is the average of 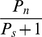 per gene where P*_n_* and P*_s_* are the numbers of synonymous and non-synonymous polymorphisms respectively. This test can be generalised to use other classes of sites as the selected test, in place of non-synonymous sites in the original MK test [33]. We calculated a using four-fold degenerate sites as the neutral control and either non-synonymous sites (for exons), intronic sites, or intergenic sites, bootstrapping (by gene) 1000 times to determine the 5^th^ and 95^th^ percentiles. Intergenic SNPs and fixed differences were assigned to a gene if they fell in the half of the intergenic region closest to the gene.

### Statistics

All statistics were performed in R (Version 2.6.0) (Ref. [68]). Tests for differences in DAF or MAF used Mann-Whitney U tests. Tests for differences in selective constraint between exon, intron, and intergenic sites within a gene used paired Mann-Whitney U tests.

## Supporting Information

Figure S1 Independent calculations of constraint within each clade. A) clade constraint calculated by requiring fixed relative branch lengths, and including long branches to the root, B) clade constraint calculated without restriction on relative branch lengths, and excluding long branches. For each clade, we show constraint in the entire genome (Full aln.), exons, intergenic regions and introns. The open symbols (triangle, circle, diamond *etc.*) show the estimates of constraint using different models, with parameters optimised using different sub-sets of the alignment (see Materials and Methods).
(0.12 MB PDF)

Figure S2 Length distributions of constrained elements at different false discovery rates. Relative number of CEs at different lengths with GERP false discovery rates. Clearly, false discoveries are biased to shorter elements. With elements >25 nt, there is little difference between FDR rates, so most elements are not false discoveries.
(0.12 MB PDF)

Figure S3 Alignment depth and gaps by annotation. A) For each annotation element (exon, intron, or intergenic region, using the *P. falciparum* annotation) we calculated the proportion positions (alignment columns) in the element that have ≥4 species represented without gaps, ‘well aligned’ regions. A minimum of four species are required for the GERP method. Similarly for intron (blue) and intergenic (black) elements. Many exons have all their length covered by well aligned regions, while very few intronic and intergenic regions are completely covered by well aligned regions B) For of each annotation we calculate the proportion of the alignment slice that is composed of gaps (rather than aligned nucleotides). While many exons contain no gap positions, many intergenic windows are ∼60% gaps.
(0.04 MB PDF)

Figure S4 Neutral rate variation across Plasmodium genomes. Evidence for long-term variation in the neutral rate across Plasmodium genomes. A) The scaling parameter (rate estimate as a proportion of the entire alignment tree length) was calculated for FFD sites falling within 40kb windows of the alignment (top left panel), and for 40kb windows of a shuffled alignment (shuffled by columns). Only windows containing at least 60 FFD sites were used. The distribution of real values is clearly stochastically wider than that from the shuffled alignments. B) FFD rates and intronic rates falling within 40kb windows of the alignment are correlated. Since many intronic sites are free to vary at close to the neutral rate, this supports the hypothesis of long-term variation in the neutral rate across Plasmodium genomes.
(0.10 MB PDF)

Figure S5 Constraint with long and short consensus. We used the annotations from *P. falciparum*, *P. knowlesi* and *P. yoelii* to identify exon, intron and intergenic regions in the alignment. These annotations are not always consistent, and so intron and intergenic start and end points may be defined in up to three locations. We defined the long consensus as the longest start – end coordinates of an intergenic/intronic region, and the short consensus as the shortest start – end coordinates. Constraint estimates near intron (upper panels) and intergenic (lower panels) boundaries for the long consensus (blue) and short consensus (black) do not differ markedly.
(0.03 MB PDF)

Table S1 Divergence and constraint estimates. A. Divergence estimates from Hyphy maximum-likelihood analysis using the 6 models (see Materials and Methods). B. Within-clade annotations analysis included regions commonly annotated by *P. knowlesi* and *P. vivax* for the primate clade, *P. yoelii*, *P. berghei* and *P. chabaudi* for the rodent clade, and only *P. falciparum* for the ape clade.
(0.18 MB DOC)

Table S2 Numbers of constrained sites in *P. falciparum* and *P. knowlesi* genomes. We define the depth of alignment required to quantify as a ‘well aligned’ site (number of species aligned). * Constraint is estimate from the Ape clade for *P. falciparum* and the Primate clade for *P. knowlesi*, using HKY85 model (with parameters estimated on all alignment positions).
(0.05 MB DOC)

Table S3 Selective constraint in CE regions. Estimates of constraint in CE regions within exons, intergenic regions (IGR) and introns and these regions excluding CE regions. Data sets used for estimating the parameters of the maximum-likelihood model are the complete alignment (‘full align’), the rapidly diverging very AT rich regions (introns, intergenic regions and FFD sites, ‘high div’) and exons.
(0.04 MB DOC)

Table S4 Alveolate genomes searched with tblastx.
(0.04 MB DOC)

Table S5 SNP calls and SNP false positive rate. A. SNP calls. B. Estimation of false positive rate for SNP calls in chromosome 12. C. Assessing the error rate of allele calling using MAQ-aligned Illumina reads from PCR-resequencing.
(0.09 MB DOC)

Table S6 *P. falciparum*polymorphism statistics. Statistics describing *P. falciparum* polymorphism. Estimates are median values from 100 kb windows, calculated using Variscan (see Materials and Methods). Columns are: Length (the total number of nucleotide positions considered), Polym (the number of polymorphic sites), Polym/Length (the number of polymorphic sites/kb considered), π (the average pairwise diversity between all species), Θ (Watterson's theta, a normalised measure of segregating (polymorphic) sites) and D (Tajima's D, an estimate of the discrepancy between π and Θ). * Values are ×10^−3^.
(0.05 MB DOC)

Table S7 Mann-Whitney Tests comparing derived allele frequency (DAF) and minor allele frequency in different annotations of the genome. A. All genes. Tests are one-sided Mann-Whitney U Tests of MAF/DAF in annotation 1 against annotation 2. All tests are shown, sorted by P-value. The Bonferonni P-value = 0.05/12 = 0.0041667. Tests with P-values less than this Bonferonni-adjusted P-value are shown in bold. B. Excluding Var, Rifin and Stevor genes. All combinations of tests were performed, as above (exon vs intron, exon vs intergenic, DAF/MAF, greater/less). Only tests with P values<0.05 are shown. Tests with P-values less than the Bonferonni-adjusted P-value of 0.0041667 are shown in bold. C. Var, Rifin and Stevor genes only. All combinations of tests were performed, as above (exon vs intron, exon vs intergenic, DAF/MAF, greater/less). Only tests with P values<0.05 are shown.
(0.06 MB DOC)

Table S8GO-Slim Groups with an excess high frequency alleles. For all 23 GO categories, we compared the DAF of all SNPs within genes the GO-Slim category to all other SNPs using a two-tailed Mann-Whitney U test. We repeated the same analysis using minor allele frequencies. Since we conducted 46 tests the Bonferonni-corrected threshold is 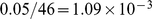. Only categories with P values <0.05 are shown.(0.05 MB DOC)

Text S1 (0.41 MB DOC)
