# Supplementary material for: Long- and Short-Term Selective Forces on Malaria Parasite Genomes
Source: PLoS Genet. 2010 Sep 9;6(9):e1001099. doi: 10.1371/journal.pgen.1001099 (PMC2936524; doi:10.1371/journal.pgen.1001099)
Supplement: Figure S1 — Independent calculations of constraint within each clade. A) clade constraint calculated by requiring fixed relative branch lengths, and including long branches to the root, B) clade constraint calculated without restriction on relative branch lengths, and excluding long branches. For each clade, we show constraint in the entire genome (Full aln.), exons, intergenic regions and introns. The open symbols (triangle, circle, diamond etc.) show the estimates of constraint using different models, with parameters optimised using different sub-sets of the alignment (see Materials and Methods). (0.12 MB PDF) [file pgen.1001099.s001.pdf]

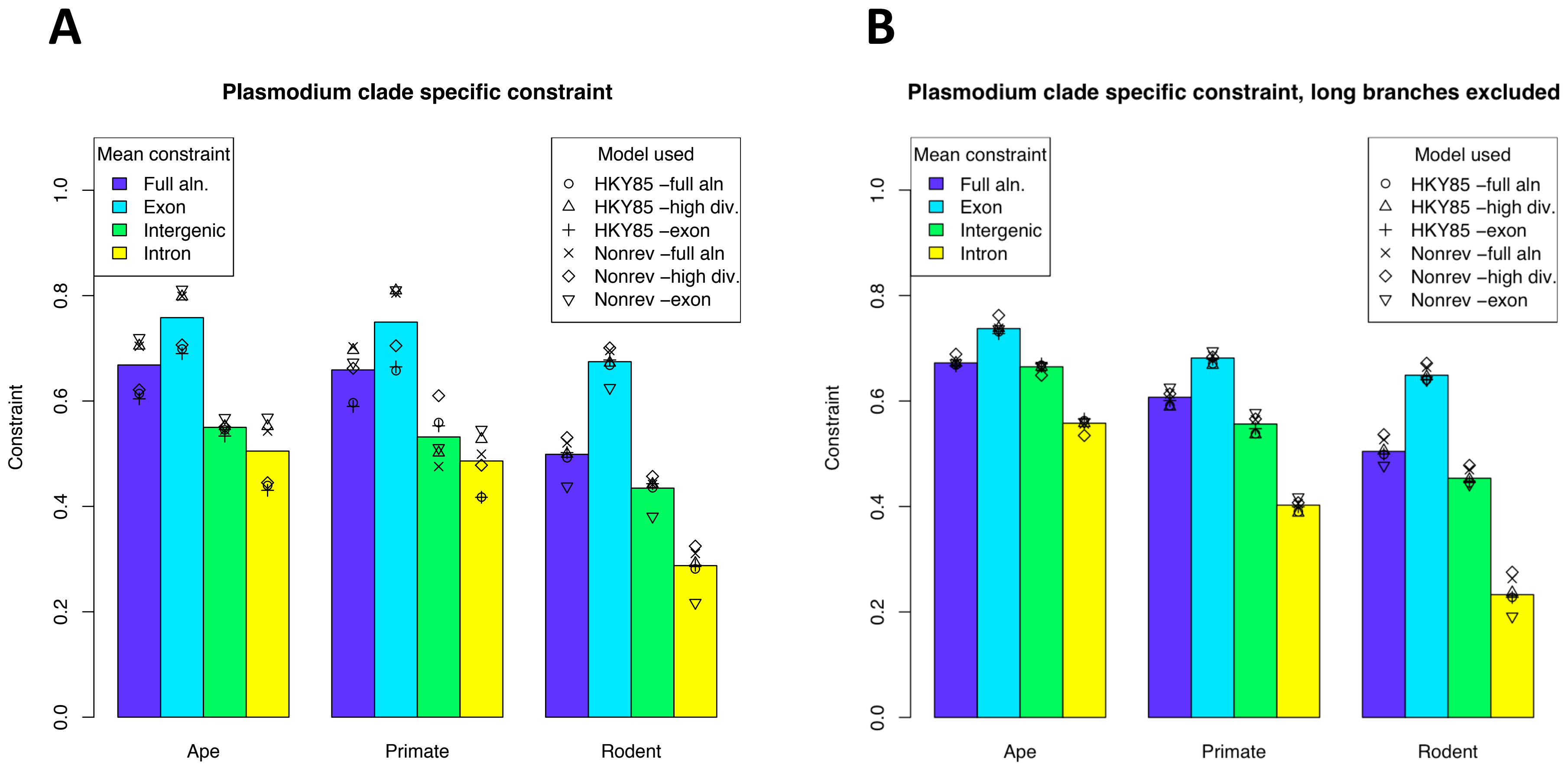
A
Plasmodium clade specific constraint
Mean constraint
Full aln.
Exon
Intergenic
Intron
Model used
HKY85 –full aln
HKY85 –high div.
HKY85 –exon
Nonrev –full aln
Nonrev –high div.
Nonrev –exon
Constraint
0.0
0.2
0.4
0.6
0.8
1.0
Ape
Primate
Rodent
B
Plasmodium clade specific constraint, long branches excluded
Mean constraint
Full aln.
Exon
Intergenic
Intron
Model used
HKY85 –full aln
HKY85 –high div.
HKY85 –exon
Nonrev –full aln
Nonrev –high div.
Nonrev –exon
Constraint
0.0
0.2
0.4
0.6
0.8
1.0
Ape
Primate
Rodent
