# Supplementary figures and images for: Long- and Short-Term Selective Forces on Malaria Parasite Genomes
Source: PLoS Genet. 2010 Sep 9;6(9):e1001099. doi: 10.1371/journal.pgen.1001099 (PMC2936524; doi:10.1371/journal.pgen.1001099)

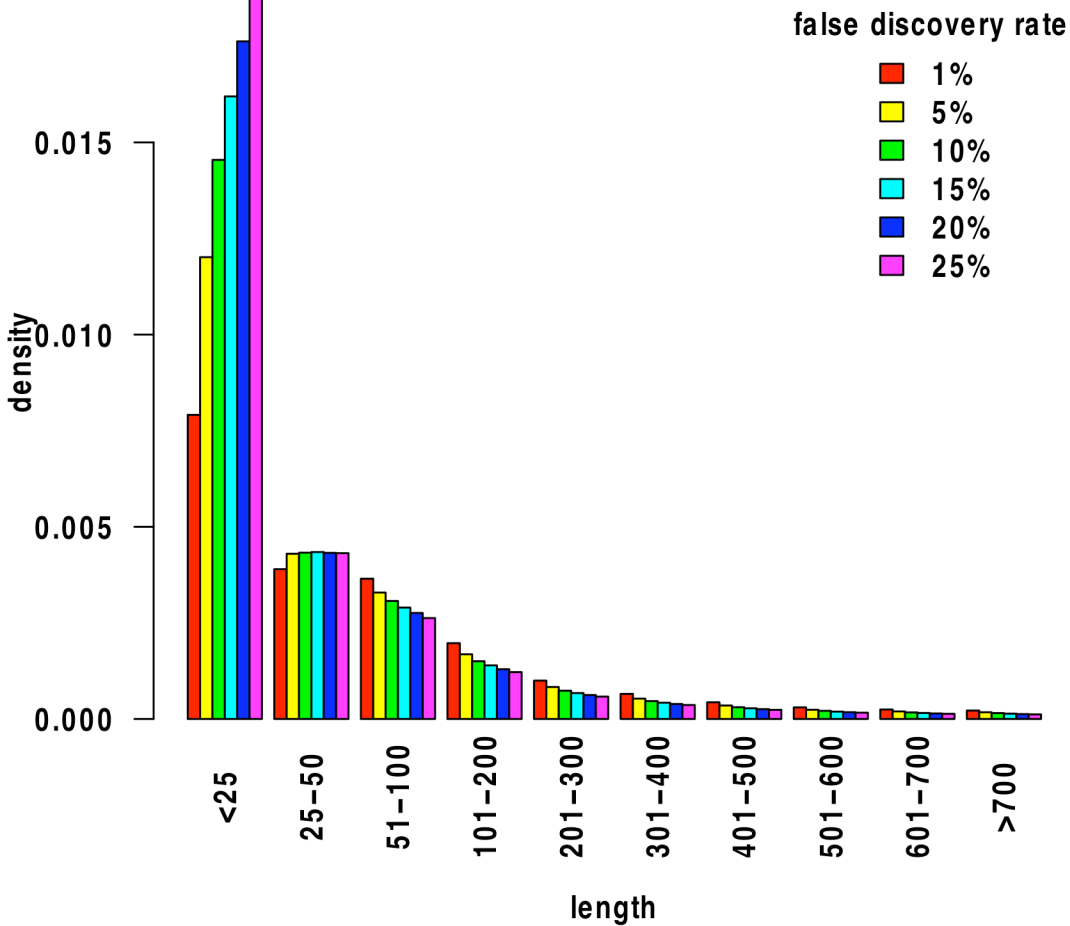

Supplement: Figure S2 — Length distributions of constrained elements at different false discovery rates. Relative number of CEs at different lengths with GERP false discovery rates. Clearly, false discoveries are biased to shorter elements. With elements >25 nt, there is little difference between FDR rates, so most elements are not false discoveries. (0.12 MB PDF) [file pgen.1001099.s002.pdf]

**A**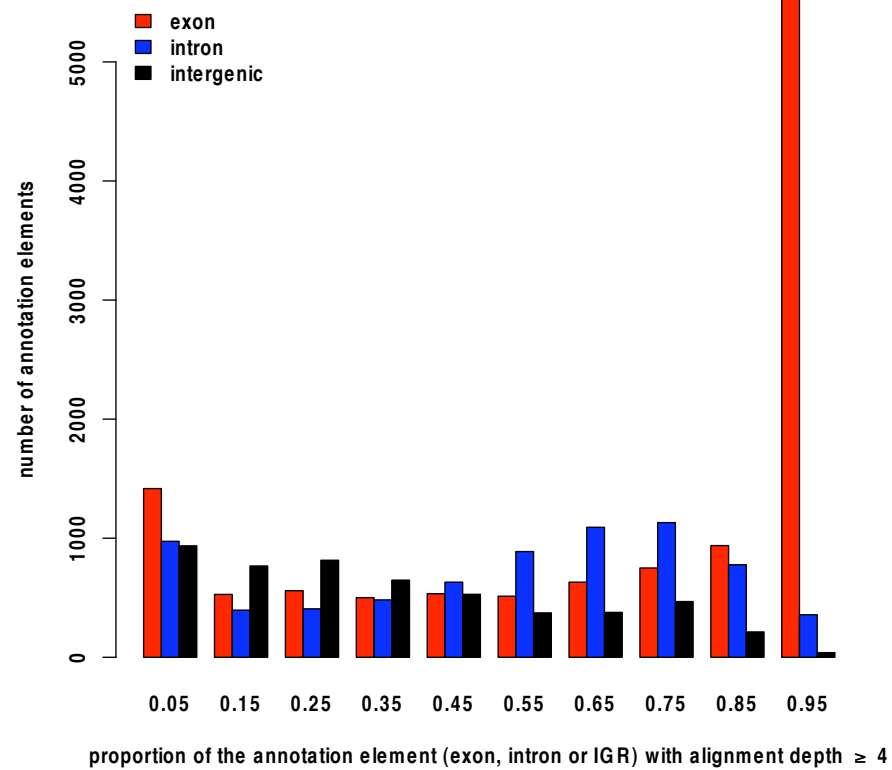**B**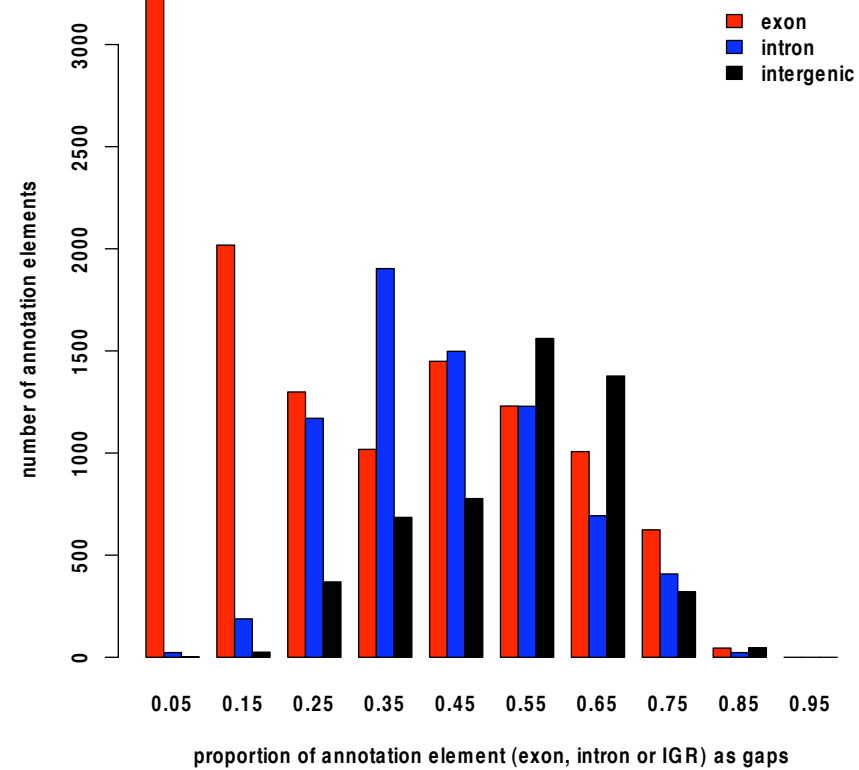

Supplement: Figure S3 — Alignment depth and gaps by annotation. A) For each annotation element (exon, intron, or intergenic region, using the P. falciparum annotation) we calculated the proportion positions (alignment columns) in the element that have ≥4 species represented without gaps, ‘well aligned’ regions. A minimum of four species are required for the GERP method. Similarly for intron (blue) and intergenic (black) elements. Many exons have all their length covered by well aligned regions, while very few intronic and intergenic regions are completely covered by well aligned regions B) For of each annotation we calculate the proportion of the alignment slice that is composed of gaps (rather than aligned nucleotides). While many exons contain no gap positions, many intergenic windows are ∼60% gaps. (0.04 MB PDF) [file pgen.1001099.s003.pdf]

**Intron (donor)**

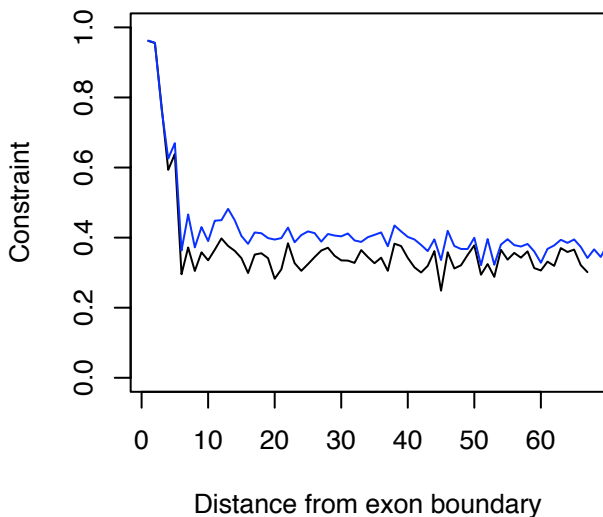

**Intron (acceptor)**

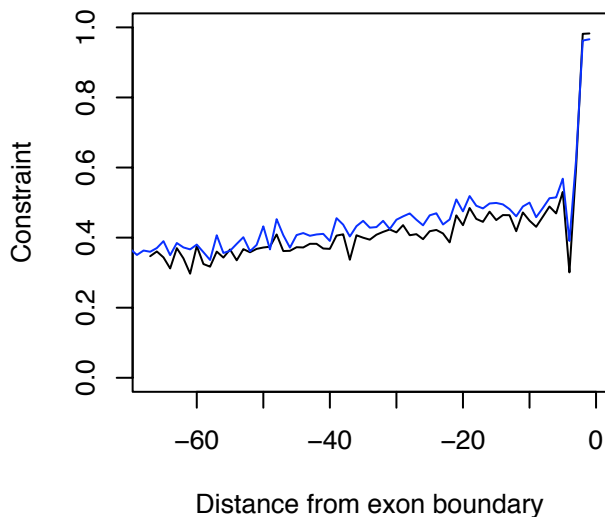

**Intergenic (promoter)**

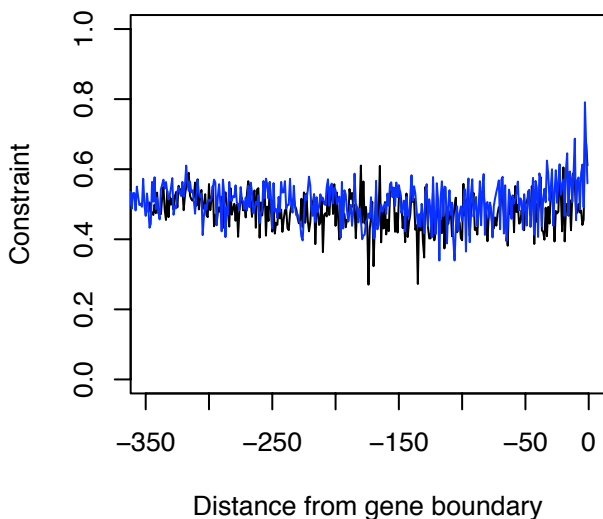

**Intergenic (terminator)**

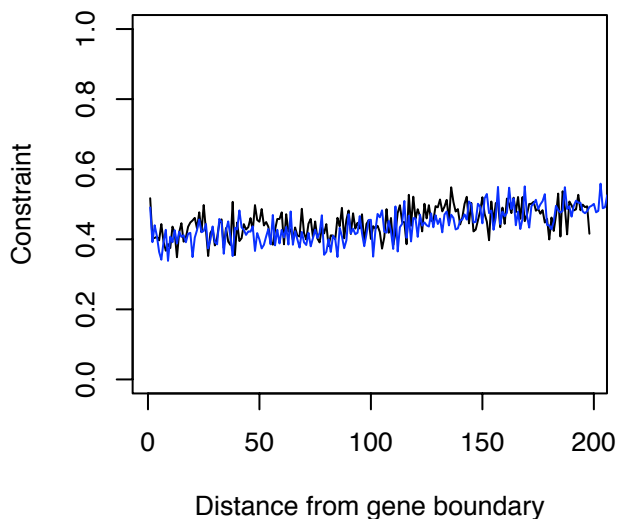

Supplement: Figure S5 — Constraint with long and short consensus. We used the annotations from P. falciparum, P. knowlesi and P. yoelii to identify exon, intron and intergenic regions in the alignment. These annotations are not always consistent, and so intron and intergenic start and end points may be defined in up to three locations. We defined the long consensus as the longest start – end coordinates of an intergenic/intronic region, and the short consensus as the shortest start – end coordinates. Constraint estimates near intron (upper panels) and intergenic (lower panels) boundaries for the long consensus (blue) and short consensus (black) do not differ markedly. (0.03 MB PDF) [file pgen.1001099.s005.pdf]
