# Supplementary material for: Long- and Short-Term Selective Forces on Malaria Parasite Genomes
Source: PLoS Genet. 2010 Sep 9;6(9):e1001099. doi: 10.1371/journal.pgen.1001099 (PMC2936524; doi:10.1371/journal.pgen.1001099)
Supplement: Figure S4 — Neutral rate variation across Plasmodium genomes. Evidence for long-term variation in the neutral rate across Plasmodium genomes. A) The scaling parameter (rate estimate as a proportion of the entire alignment tree length) was calculated for FFD sites falling within 40kb windows of the alignment (top left panel), and for 40kb windows of a shuffled alignment (shuffled by columns). Only windows containing at least 60 FFD sites were used. The distribution of real values is clearly stochastically wider than that from the shuffled alignments. B) FFD rates and intronic rates falling within 40kb windows of the alignment are correlated. Since many intronic sites are free to vary at close to the neutral rate, this supports the hypothesis of long-term variation in the neutral rate across Plasmodium genomes. (0.10 MB PDF) [file pgen.1001099.s004.pdf]

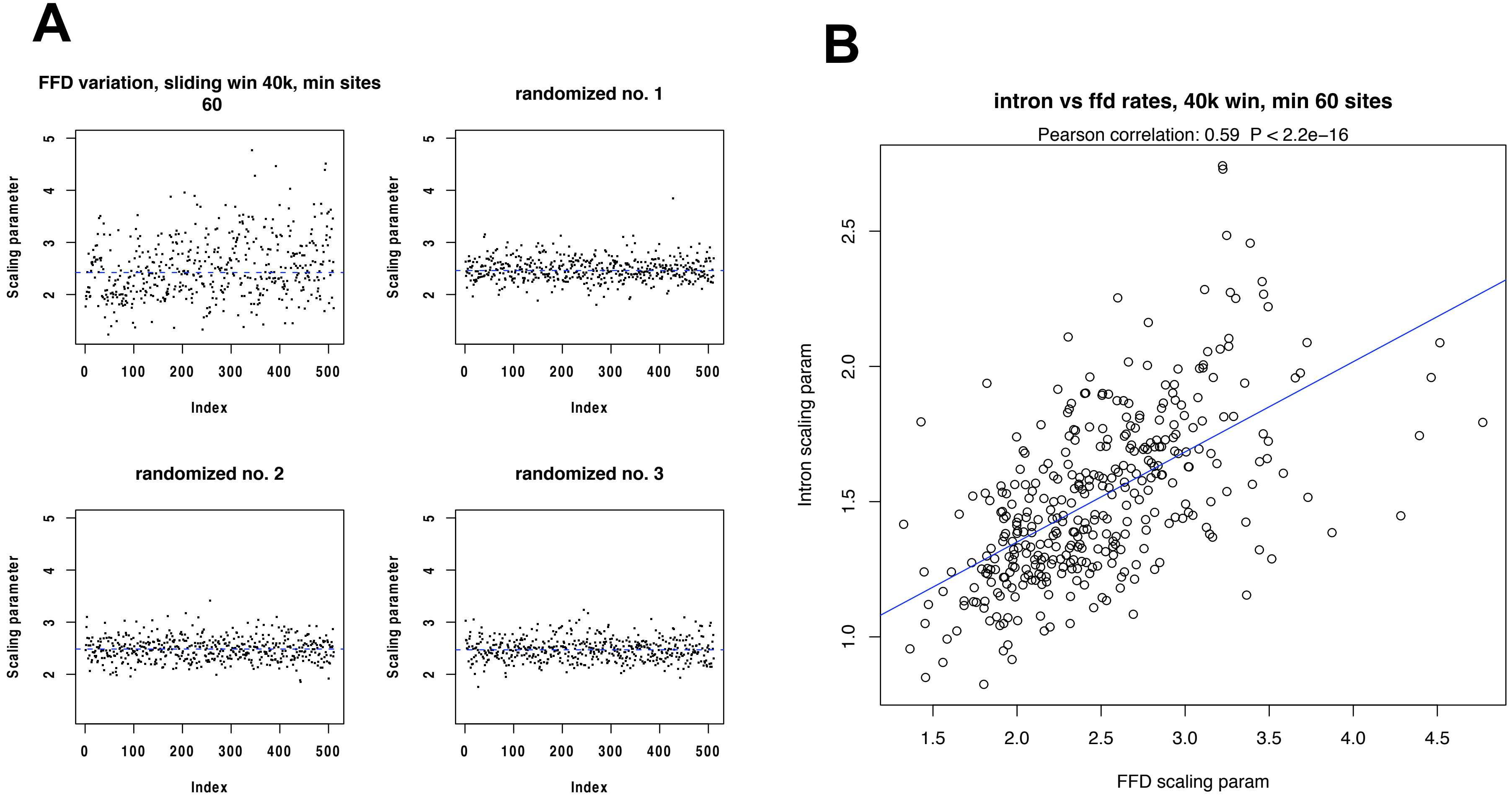
A
FFD variation, sliding win 40k, min sites 60
randomized no. 1
randomized no. 2
randomized no. 3
Scaling parameter
Index
0
100
200
300
400
500
2
3
4
5
B
intron vs ffd rates, 40k win, min 60 sites
Pearson correlation: 0.59 P < 2.2e−16
Intron scaling param
FFD scaling param
1.0
1.5
2.0
2.5
3.0
3.5
4.0
4.5
