# Supplementary material for: Long- and Short-Term Selective Forces on Malaria Parasite Genomes
Source: PLoS Genet. 2010 Sep 9;6(9):e1001099. doi: 10.1371/journal.pgen.1001099 (PMC2936524; doi:10.1371/journal.pgen.1001099)
Supplement: Table S1 — Divergence and constraint estimates. A. Divergence estimates from Hyphy maximum-likelihood analysis using the 6 models (see Materials and Methods). B. Within-clade annotations analysis included regions commonly annotated by P. knowlesi and P. vivax for the primate clade, P. yoelii, P. berghei and P. chabaudi for the rodent clade, and only P. falciparum for the ape clade. (0.18 MB DOC) [file pgen.1001099.s006.doc]

**Table S1a: Divergence estimates.**

Divergence estimates from Hyphy maximum-likelihood analysis using the 6 models (see methods).

| #Full Aln |  |  |  |  |  |  |  |  |  |  |  |  |
| --- | --- | --- | --- | --- | --- | --- | --- | --- | --- | --- | --- | --- |
| Model | PlaBer | PlaYoe | Node2 | PlaCha | Node1 | PlaViv | PlaKno | Node6 | PlaFal | PlaRei | Node9 | Scaling |
| globalHKYtree_full | 0.035 | 0.052 | 0.052 | 0.061 | 0.244 | 0.121 | 0.088 | 0.306 | 0.006 | 0.017 | 0.279 | 1.000 |
| globalHKYtree_nc | 0.034 | 0.052 | 0.052 | 0.059 | 0.240 | 0.115 | 0.087 | 0.294 | 0.006 | 0.017 | 0.277 | 1.000 |
| globalHKYtree_exon | 0.035 | 0.053 | 0.053 | 0.061 | 0.258 | 0.121 | 0.090 | 0.316 | 0.006 | 0.017 | 0.292 | 1.000 |
| local12tree_full | 0.033 | 0.051 | 0.049 | 0.060 | 0.250 | 0.159 | 0.058 | 0.228 | 0.006 | 0.016 | 0.259 | 1.000 |
| local12tree_nc | 0.033 | 0.049 | 0.048 | 0.058 | 0.240 | 0.157 | 0.057 | 0.252 | 0.006 | 0.013 | 0.201 | 1.000 |
| local12tree_exon | 0.035 | 0.052 | 0.048 | 0.066 | 0.224 | 0.175 | 0.067 | 0.237 | 0.006 | 0.017 | 0.341 | 1.000 |
|  |  |  |  |  |  |  |  |  |  |  |  |  |
| #Exon |  |  |  |  |  |  |  |  |  |  |  |  |
| Model | PlaBer | PlaYoe | Node2 | PlaCha | Node1 | PlaViv | PlaKno | Node6 | PlaFal | PlaRei | Node9 | Scaling |
| globalHKYtree_full | 0.025 | 0.037 | 0.037 | 0.044 | 0.175 | 0.087 | 0.063 | 0.219 | 0.004 | 0.012 | 0.200 | 0.716 |
| globalHKYtree_nc | 0.024 | 0.037 | 0.037 | 0.042 | 0.172 | 0.083 | 0.062 | 0.211 | 0.004 | 0.012 | 0.199 | 0.715 |
| globalHKYtree_exon | 0.025 | 0.037 | 0.037 | 0.043 | 0.182 | 0.086 | 0.064 | 0.224 | 0.004 | 0.012 | 0.206 | 0.707 |
| local12tree_full | 0.024 | 0.037 | 0.035 | 0.043 | 0.180 | 0.115 | 0.042 | 0.165 | 0.004 | 0.011 | 0.187 | 0.722 |
| local12tree_nc | 0.023 | 0.036 | 0.035 | 0.042 | 0.173 | 0.113 | 0.041 | 0.181 | 0.004 | 0.010 | 0.144 | 0.719 |
| local12tree_exon | 0.026 | 0.038 | 0.035 | 0.048 | 0.163 | 0.127 | 0.049 | 0.172 | 0.004 | 0.012 | 0.248 | 0.728 |
|  |  |  |  |  |  |  |  |  |  |  |  |  |
| #IGR |  |  |  |  |  |  |  |  |  |  |  |  |
| Model | PlaBer | PlaYoe | Node2 | PlaCha | Node1 | PlaViv | PlaKno | Node6 | PlaFal | PlaRei | Node9 | Scaling |
| globalHKYtree_full | 0.042 | 0.063 | 0.063 | 0.073 | 0.295 | 0.147 | 0.106 | 0.370 | 0.007 | 0.021 | 0.337 | 1.207 |
| globalHKYtree_nc | 0.041 | 0.062 | 0.063 | 0.072 | 0.290 | 0.140 | 0.105 | 0.356 | 0.007 | 0.021 | 0.336 | 1.211 |
| globalHKYtree_exon | 0.042 | 0.064 | 0.064 | 0.075 | 0.313 | 0.148 | 0.109 | 0.385 | 0.007 | 0.021 | 0.354 | 1.216 |
| local12tree_full | 0.040 | 0.061 | 0.059 | 0.071 | 0.299 | 0.190 | 0.070 | 0.273 | 0.007 | 0.019 | 0.310 | 1.196 |
| local12tree_nc | 0.039 | 0.060 | 0.058 | 0.070 | 0.289 | 0.189 | 0.069 | 0.303 | 0.007 | 0.016 | 0.242 | 1.204 |
| local12tree_exon | 0.042 | 0.062 | 0.057 | 0.078 | 0.267 | 0.208 | 0.080 | 0.282 | 0.007 | 0.020 | 0.406 | 1.192 |
|  |  |  |  |  |  |  |  |  |  |  |  |  |
| #Intron |  |  |  |  |  |  |  |  |  |  |  |  |
| Model | PlaBer | PlaYoe | Node2 | PlaCha | Node1 | PlaViv | PlaKno | Node6 | PlaFal | PlaRei | Node9 | Scaling |
| globalHKYtree_full | 0.050 | 0.076 | 0.075 | 0.088 | 0.354 | 0.176 | 0.127 | 0.444 | 0.008 | 0.025 | 0.405 | 1.449 |
| globalHKYtree_nc | 0.049 | 0.075 | 0.075 | 0.086 | 0.347 | 0.167 | 0.126 | 0.426 | 0.008 | 0.025 | 0.402 | 1.447 |
| globalHKYtree_exon | 0.051 | 0.077 | 0.077 | 0.090 | 0.377 | 0.178 | 0.132 | 0.463 | 0.009 | 0.025 | 0.427 | 1.464 |
| local12tree_full | 0.049 | 0.074 | 0.072 | 0.087 | 0.365 | 0.232 | 0.085 | 0.333 | 0.009 | 0.023 | 0.378 | 1.460 |
| local12tree_nc | 0.048 | 0.073 | 0.071 | 0.085 | 0.352 | 0.231 | 0.084 | 0.369 | 0.009 | 0.020 | 0.295 | 1.466 |
| local12tree_exon | 0.051 | 0.076 | 0.070 | 0.096 | 0.328 | 0.255 | 0.098 | 0.346 | 0.009 | 0.024 | 0.498 | 1.462 |
|  |  |  |  |  |  |  |  |  |  |  |  |  |
| #FFD |  |  |  |  |  |  |  |  |  |  |  |  |
| Model | PlaBer | PlaYoe | Node2 | PlaCha | Node1 | PlaViv | PlaKno | Node6 | PlaFal | PlaRei | Node9 | Scaling |
| globalHKYtree_full | 0.084 | 0.127 | 0.126 | 0.148 | 0.594 | 0.295 | 0.213 | 0.744 | 0.014 | 0.042 | 0.679 | 2.432 |
| globalHKYtree_nc | 0.082 | 0.125 | 0.125 | 0.143 | 0.580 | 0.279 | 0.210 | 0.712 | 0.014 | 0.041 | 0.671 | 2.418 |
| globalHKYtree_exon | 0.087 | 0.132 | 0.131 | 0.153 | 0.641 | 0.302 | 0.224 | 0.787 | 0.015 | 0.043 | 0.725 | 2.487 |
| local12tree_full | 0.081 | 0.123 | 0.120 | 0.145 | 0.608 | 0.387 | 0.141 | 0.556 | 0.015 | 0.038 | 0.631 | 2.434 |
| local12tree_nc | 0.080 | 0.121 | 0.118 | 0.142 | 0.588 | 0.385 | 0.140 | 0.616 | 0.015 | 0.033 | 0.492 | 2.449 |
| local12tree_exon | 0.086 | 0.128 | 0.117 | 0.161 | 0.550 | 0.428 | 0.165 | 0.580 | 0.015 | 0.041 | 0.836 | 2.451 |


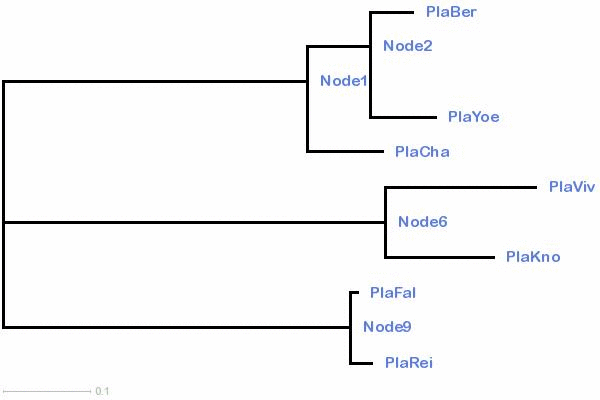


**Node definitions for Table S1a.**

**Table S1b. Constraint estimates**

* Within-clade annotations analysis included regions commonly annotated by P. knowlesi and P. vivax for the primate clade, P. yoelii, P. berghei and P. chabaudi for the rodent clade, and only P. falciparum for the ape clade.

| #Regions for constrain estimate.  Model-parameterisation region used | Incl. long branches | Incl. long branch | Incl. long branch | Incl. long branch | Excl. long branch | Excl. long branch | Excl. long branch | Excl. long branch, using only within-clade annotations* | Excl. long branch, using only within-clade annotations* | Excl. long branch, using only within-clade annotations* |
| --- | --- | --- | --- | --- | --- | --- | --- | --- | --- | --- |
| #Full Aln | Full tree | Ape | Primate | Rodent | Ape | Primate | Rodent | Ape | Primate | Rodent |
| HKY85-full | 0.589 | 0.614 | 0.597 | 0.492 | 0.668 | 0.591 | 0.499 | 0.668 | 0.591 | 0.488 |
| HKY85-high div. | 0.586 | 0.705 | 0.696 | 0.500 | 0.672 | 0.589 | 0.506 |  |  |  |
| HKY85-exon | 0.598 | 0.604 | 0.590 | 0.502 | 0.667 | 0.601 | 0.499 |  |  |  |
| Nonrev-full | 0.589 | 0.704 | 0.703 | 0.520 | 0.672 | 0.614 | 0.527 |  |  |  |
| Nonrev-high div. | 0.592 | 0.621 | 0.662 | 0.531 | 0.689 | 0.614 | 0.536 |  |  |  |
| Nonrev-exon | 0.592 | 0.720 | 0.673 | 0.438 | 0.672 | 0.626 | 0.478 |  |  |  |
| Mean | 0.591 | 0.661 | 0.654 | 0.497 | 0.673 | 0.606 | 0.507 |  |  |  |
|  |  |  |  |  |  |  |  |  |  |  |
| #Exon |  |  |  |  |  |  |  |  |  |  |
| HKY85-full | 0.706 | 0.699 | 0.657 | 0.668 | 0.731 | 0.671 | 0.640 | 0.721 | 0.869 | 0.841 |
| HKY85-high div. | 0.704 | 0.798 | 0.810 | 0.672 | 0.738 | 0.668 | 0.646 |  |  |  |
| HKY85-exon | 0.716 | 0.690 | 0.665 | 0.678 | 0.728 | 0.679 | 0.641 |  |  |  |
| Nonrev-full | 0.703 | 0.799 | 0.805 | 0.696 | 0.738 | 0.685 | 0.664 |  |  |  |
| Nonrev-high div. | 0.706 | 0.707 | 0.705 | 0.701 | 0.762 | 0.684 | 0.672 |  |  |  |
| Nonrev-exon | 0.703 | 0.812 | 0.810 | 0.625 | 0.736 | 0.695 | 0.640 |  |  |  |
| Mean | 0.706 | 0.751 | 0.742 | 0.673 | 0.739 | 0.680 | 0.650 |  |  |  |
|  |  |  |  |  |  |  |  |  |  |  |
| #IGR |  |  |  |  |  |  |  |  |  |  |
| HKY85-full | 0.504 | 0.545 | 0.559 | 0.435 | 0.668 | 0.538 | 0.445 | 0.704 | 0.685 | 0.661 |
| HKY85-high div. | 0.499 | 0.552 | 0.502 | 0.442 | 0.665 | 0.536 | 0.451 |  |  |  |
| HKY85-exon | 0.511 | 0.533 | 0.553 | 0.443 | 0.670 | 0.548 | 0.446 |  |  |  |
| Nonrev-full | 0.509 | 0.545 | 0.476 | 0.442 | 0.664 | 0.564 | 0.470 |  |  |  |
| Nonrev-high div. | 0.508 | 0.551 | 0.610 | 0.457 | 0.649 | 0.566 | 0.478 |  |  |  |
| Nonrev-exon | 0.514 | 0.568 | 0.511 | 0.381 | 0.667 | 0.578 | 0.442 |  |  |  |
| Mean | 0.507 | 0.549 | 0.535 | 0.433 | 0.664 | 0.555 | 0.455 |  |  |  |
|  |  |  |  |  |  |  |  |  |  |  |
| #Intron |  |  |  |  |  |  |  |  |  |  |
| HKY85-full | 0.404 | 0.440 | 0.418 | 0.281 | 0.563 | 0.390 | 0.228 | 0.682 | 0.557 | 0.413 |
| HKY85-high div. | 0.401 | 0.552 | 0.528 | 0.292 | 0.558 | 0.388 | 0.237 |  |  |  |
| HKY85-exon | 0.411 | 0.431 | 0.417 | 0.288 | 0.566 | 0.400 | 0.228 |  |  |  |
| Nonrev-full | 0.400 | 0.543 | 0.499 | 0.311 | 0.557 | 0.405 | 0.263 |  |  |  |
| Nonrev-high div. | 0.401 | 0.445 | 0.478 | 0.325 | 0.535 | 0.407 | 0.275 |  |  |  |
| Nonrev-exon | 0.404 | 0.569 | 0.546 | 0.217 | 0.561 | 0.418 | 0.192 |  |  |  |
| Mean | 0.404 | 0.497 | 0.481 | 0.286 | 0.556 | 0.401 | 0.237 |  |  |  |
