# Supplementary material for: Long- and Short-Term Selective Forces on Malaria Parasite Genomes
Source: PLoS Genet. 2010 Sep 9;6(9):e1001099. doi: 10.1371/journal.pgen.1001099 (PMC2936524; doi:10.1371/journal.pgen.1001099)
Supplement: Table S2 — Numbers of constrained sites in P. falciparum and P. knowlesi genomes. We define the depth of alignment required to quantify as a ‘well aligned’ site (number of species aligned). * Constraint is estimate from the Ape clade for P. falciparum and the Primate clade for P. knowlesi, using HKY85 model (with parameters estimated on all alignment positions). (0.05 MB DOC) [file pgen.1001099.s007.doc]

**Table S2. Numbers of Constrained Sites in *P. falciparum* and *P. knowlesi* Genomes**

We define the depth of alignment required to quantify as a ‘well aligned’ site (number of species aligned). * Constraint is estimate from the Ape clade for *P. falciparum* and the Primate clade for *P. knowlesi*, using HKY85 model (with parameters estimated on all alignment positions).

| Species | Depth | Region | Well aligned Sites (kb) | Constraint* | Constrained Sites (kb) | Proportion vs. exonic CN |
| --- | --- | --- | --- | --- | --- | --- |
| P. falciparum | 2 | genome | 16,492 | 0.59 | 9,710 |  |
|  |  | exon | 9,726 | 0.71 | 6,864 | 1.00 |
|  |  | intron | 1,115 | 0.50 | 562 | 0.08 |
|  |  | intergenic | 5,581 | 0.51 | 2,832 | 0.41 |
|  |  |  |  |  |  |  |
| P. falciparum | 4 | genome | 10,160 | 0.59 | 5,982 |  |
|  |  | exon | 6,751 | 0.71 | 4,765 | 1.00 |
|  |  | intron | 766 | 0.50 | 386 | 0.08 |
|  |  | intergenic | 2,611 | 0.51 | 1,325 | 0.28 |
|  |  |  |  |  |  |  |
| P. falciparum | 6 | genome | 4,687 | 0.59 | 2,760 |  |
|  |  | exon | 3,592 | 0.71 | 2,535 | 1.00 |
|  |  | intron | 319 | 0.50 | 161 | 0.06 |
|  |  | intergenic | 761 | 0.51 | 386 | 0.15 |
|  |  |  |  |  |  |  |
| P. knowlesi | 4 | genome | 10,644 | 0.60 | 6,354 |  |
|  |  | exon | 6,783 | 0.66 | 4,459 | 1.00 |
|  |  | intron | 799 | 0.42 | 334 | 0.07 |
|  |  | intergenic | 3,058 | 0.56 | 1,710 | 0.38 |
