# Supplementary material for: Long- and Short-Term Selective Forces on Malaria Parasite Genomes
Source: PLoS Genet. 2010 Sep 9;6(9):e1001099. doi: 10.1371/journal.pgen.1001099 (PMC2936524; doi:10.1371/journal.pgen.1001099)
Supplement: Table S3 — Selective constraint in CE regions. Estimates of constraint in CE regions within exons, intergenic regions (IGR) and introns and these regions excluding CE regions. Data sets used for estimating the parameters of the maximum-likelihood model are the complete alignment (‘full align’), the rapidly diverging very AT rich regions (introns, intergenic regions and FFD sites, ‘high div’) and exons. (0.04 MB DOC) [file pgen.1001099.s008.doc]

**Table S3. Selective constraint in CE regions**

Estimates of constraint in CE regions within exons, intergenic regions (IGR) and introns and these regions excluding CE regions.

Data sets used for estimating the parameters of the maximum-likelihood model are the complete alignment (‘full align’), the rapidly diverging very AT rich regions (introns, intergenic regions and FFD sites, ‘high div’) and exons.

| Model/data set | Exon CEs | Exon excluding CEs | IGR CEs | IGR excluding CEs | Intron CEs | Intron excluding CEs |
| --- | --- | --- | --- | --- | --- | --- |
| HKY/full align. | 0.780 | 0.504 | 0.723 | 0.497 | 0.701 | 0.400 |
| HKY/high div. | 0.778 | 0.504 | 0.718 | 0.493 | 0.699 | 0.397 |
| HKY/exon | 0.788 | 0.514 | 0.731 | 0.504 | 0.711 | 0.407 |
| Nonrev/full align. | 0.781 | 0.497 | 0.736 | 0.503 | 0.707 | 0.403 |
| Nonrev/high div. | 0.783 | 0.505 | 0.734 | 0.503 | 0.709 | 0.404 |
| Nonrev/exon | 0.780 | 0.491 | 0.743 | 0.507 | 0.709 | 0.407 |
| Average | 0.782 | 0.503 | 0.731 | 0.501 | 0.706 | 0.403 |
