# Supplementary material for: Long- and Short-Term Selective Forces on Malaria Parasite Genomes
Source: PLoS Genet. 2010 Sep 9;6(9):e1001099. doi: 10.1371/journal.pgen.1001099 (PMC2936524; doi:10.1371/journal.pgen.1001099)
Supplement: Table S4 — Alveolate genomes searched with tblastx. (0.04 MB DOC) [file pgen.1001099.s009.doc]

**Table S4. Alveolate genomes searched with tblastx**

| Species | Source (version) |
| --- | --- |
| *Paramecium tetraurelia* | Refseq accession NC_006058.1 |
| *Tetrahymena thermophila* | Refseq accessions NZ_AAGF0100* |
| *Theileria parva* | Refseq accessions NC_007345.1, NC_007344.1, NC_011005.1, NC_007758.1 |
| *Theileria annulata* | Refseq accessions NC_011098.1, NC_011100.1, NC_011129.1, NC_011099.1 |
| *Cryptosporidium parvum* | Refseq accessions NC_006987.1, , NC_006986.1, NC_006983.1, NC_006982.1, NC_006985.1, NC_006980.1, NC_006984.1, NC_006981.1 |
| *Cryptosporidium hominis* | Genbank accessions AAEL01000001:AAEL01001422[PACC], obtained from CryptoDB, Release 4.0. |
| *Babesia bovis* | Refseq accessions NC_010575.1, NC_010574.1, NC_011395.1, NC_009902.1 |
| *Toxoplasma gondii* | Obtained from ToxoDB Release 5.0. http://toxodb.org/common/downloads/release-5.0/Tgondii/TgondiiME49Genomic_ToxoDB-5.0.fasta |
| *Neospora caninum* | Obtained from ftp://ftp.sanger.ac.uk/pub/pathogens/Neospora/caninum/ |
|  | Shotgun assembly 31/10/08, file 2nd_run_031008.tar.gz |
| *Eimeria tenella* | Assembly 08/05/2007 obtained from ftp://ftp.sanger.ac.uk/pub/pathogens/Eimeria/tenella/genome/assemblies/ |
|  | file assembly_2007_05_08.gz |
