# Supplementary material for: Long- and Short-Term Selective Forces on Malaria Parasite Genomes
Source: PLoS Genet. 2010 Sep 9;6(9):e1001099. doi: 10.1371/journal.pgen.1001099 (PMC2936524; doi:10.1371/journal.pgen.1001099)
Supplement: Table S5 — SNP calls and SNP false positive rate. A. SNP calls. B. Estimation of false positive rate for SNP calls in chromosome 12. C. Assessing the error rate of allele calling using MAQ-aligned Illumina reads from PCR-resequencing. (0.09 MB DOC) [file pgen.1001099.s010.doc]

**Table S5a. SNP Calls**

| Isolate | Reads | ssaha_SNP calls* | SNP calls derived** | Total SNPs discovered | SNPs passed | Proportion SNPs passed | Coverage (%)*** | Isolate source | Continent | Produced by | Notes |
| --- | --- | --- | --- | --- | --- | --- | --- | --- | --- | --- | --- |
| Total | 709,088 | 56,169 | 55,421 | 111,590 | 69,805 | 0.63 | - | - | - | - |  |
| R033 | 26,435 | 2,405 | 9,076 | 11,481 | 2,826 | 0.25 | 19 | Ghana | Africa | Broad |  |
| D6 | 30,115 | 3,800 | 8,916 | 12,716 | 4,178 | 0.33 | 25 | Sierra Leone | Africa | Broad |  |
| SEN3404 | 32,139 | 4,844 | 8,670 | 13,514 | 5,297 | 0.39 | 31 | Senegal | Africa | Broad |  |
| PFCLIN | 225,550 | 22,972 | 2,407 | 25,379 | 21,182 | 0.83 | 79 | Ghana | Africa | WTSI |  |
| 7G8 | 20,850 | 1,765 | 8,098 | 9,863 | 2,012 | 0.20 | 14 | Brazil | America | Broad |  |
| HB3 | 28,421 | 4,810 | 8,037 | 12,847 | 5,224 | 0.41 | 25 | Honduras | America | Broad |  |
| SANTA LUCIA | 28,659 | 3,325 | 8,851 | 12,176 | 3,855 | 0.32 | 24 | El Salvador | America | Broad |  |
| IT | 186,137 | 25,016 | 2,754 | 27,770 | 23,386 | 0.84 | 70 | Brazil | America | WTSI |  |
| DD2 | 15,489 | 2,581 | 6,123 | 8,704 | 2,943 | 0.34 | 12 | Indochina/Laos | Asia | Broad |  |
| V1/S | 16,576 | 1,582 | 7,587 | 9,169 | 1,806 | 0.20 | 11 | Vietnam | Asia | Broad |  |
| K1 | 31,621 | 4,374 | 9,788 | 14,162 | 4,980 | 0.35 | 28 | Thailand | Asia | Broad |  |
| FCC 2 | 38,995 | 5,678 | 9,871 | 15,549 | 6,387 | 0.41 | 33 | China | Asia | Broad |  |
| D10 | 28,101 | 4,138 | 9,809 | 13,947 | 4,719 | 0.34 | 24 | Papua New Guineau | Oceanea | Broad |  |
|  |  |  |  |  |  |  |  |  |  |  |  |
| P. reichenowi | 47,832 | 189,416 | 7,196 | 196,612 | 190,631 | 0.97 | 38 | Democratic Republic of the Congo | Africa | WTSI | Outgroup |

* Raw SNP calls produced by ssaha_pileup

** SNP calls derived from alignment lookup (see methods)

** Coverage is percent of the genome covered by at least two-depth read alignment

**Table S5b. Estimation of false positive rate for SNP calls in chromosome 12.**

| Isolate | Reads* | ssaha_SNP calls* | SNP calls derived | Total SNPs discovered | SNPs passed | Proportion SNPs passed | Coverage (%) | Estimated FDR (%)** | Expected number false SNPs given coverage | Estimated FDR given coverage (%)*** | Notes |
| --- | --- | --- | --- | --- | --- | --- | --- | --- | --- | --- | --- |
| 3D7 | 30,840 | 805 | 360 | 1,165 | 47 | 0.04 | 95 | 100.00 | 47.00 | 100.00 | Control |
| PFCLIN | 22,275 | 2346 | 290 | 2636 | 2160 | 0.82 | 79 | 2.18 | 39.08 | 1.81 |  |
| IT | 18,005 | 2784 | 300 | 3084 | 2583 | 0.84 | 70 | 1.82 | 34.63 | 1.34 |  |
| V1/S | 14,895 | 260 | 869 | 1129 | 322 | 0.29 | 11 | 14.60 | 5.44 | 1.69 |  |

* Reads aligned to chr 12 only

** Here we assume that all 3D7 SNPs are false discoveries

*** Takes coverage into account. Eg: since V1/S has only 11% coverage, we expect only 11/95 as many false calls as 3D7 shows (11/95 x 47).

**Table S5c. Assessing the error rate of allele calling using MAQ-aligned Illumina reads from PCR-resequencing**

| Isolate | Sites | Minimum illumina calls | Total calls compared | Same 1st ill call | Same 2nd ill call | Different | Error (1st illumina allele match only) | Error (1st or 2nd illumina allele match) | Notes |
| --- | --- | --- | --- | --- | --- | --- | --- | --- | --- |
| IT | all | 10 | 3757 | 3677 | 30 | 50 | 2.13 | 1.33 |  |
| PFCLIN | all | 10 | 3967 | 3912 | 36 | 19 | 1.39 | 0.48 |  |
| IT | all | 20 | 3475 | 3419 | 24 | 32 | 1.61 | 0.92 |  |
| PFCLIN | all | 20 | 3615 | 3569 | 31 | 15 | 1.27 | 0.41 |  |
| IT | exon | 10 | 3360 | 3305 | 28 | 27 | 1.64 | 0.80 |  |
| PFCLIN | exon | 10 | 3450 | 3407 | 18 | 25 | 1.25 | 0.72 |  |
| IT | FFD | 10 | 498 | 491 | 2 | 5 | 1.41 | 1.00 |  |
| PFCLIN | FFD | 10 | 474 | 467 | 2 | 5 | 1.48 | 1.05 |  |
| IT | intergenic | 10 | 256 | 234 | 1 | 21 | 8.59 | 8.20 | Significantly higher error rate than IT exons (Chi squared test, P < 2.2e-16) |
| PFCLIN | intergenic | 10 | 362 | 354 | 0 | 8 | 2.21 | 2.21 | Significantly higher error rate than PFCLIN exons (Chi squared test, P = 0.005891) |
| IT | intron | 10 | 141 | 138 | 1 | 2 | 2.13 | 1.42 |  |
| PFCLIN | intron | 10 | 155 | 151 | 1 | 3 | 2.58 | 1.94 |  |
| IT | nonsynon | 10 | 2259 | 2223 | 15 | 21 | 1.59 | 0.93 |  |
| PFCLIN | nonsynon | 10 | 2291 | 2270 | 8 | 13 | 0.92 | 0.57 |  |
