# Supplementary material for: Long- and Short-Term Selective Forces on Malaria Parasite Genomes
Source: PLoS Genet. 2010 Sep 9;6(9):e1001099. doi: 10.1371/journal.pgen.1001099 (PMC2936524; doi:10.1371/journal.pgen.1001099)
Supplement: Table S6 — P. falciparumpolymorphism statistics. Statistics describing P. falciparum polymorphism. Estimates are median values from 100 kb windows, calculated using Variscan (see Materials and Methods). Columns are: Length (the total number of nucleotide positions considered), Polym (the number of polymorphic sites), Polym/Length (the number of polymorphic sites/kb considered), π (the average pairwise diversity between all species), Θ (Watterson's theta, a normalised measure of segregating (polymorphic) sites) and D (Tajima's D, an estimate of the discrepancy between π and Θ). * Values are ×10−3. (0.05 MB DOC) [file pgen.1001099.s011.doc]

**Table S6. *P. falciparum* Polymorphism Statistics**

Statistics describing *P. falciparum* polymorphism. Estimates are median values from 100 kb windows, calculated using Variscan (see methods). Columns are: Length (the total number of nucleotide positions considered), Polym (the number of polymorphic sites), Polym/Length (the number of polymorphic sites/kb considered), π (the average pairwise diversity between all species), Θ (Watterson’s theta, a normalised measure of segregating (polymorphic) sites) and D (Tajima’s D, an estimate of the discrepancy between π and Θ). * Values are x10^-3^.

| Annotation | Length | Polym | Polym/Length * | π* | Θ* | D |
| --- | --- | --- | --- | --- | --- | --- |
| Genome | 12,445,183 | 29,201 | 2.35 | 0.99 | 1.03 | -0.35 |
|  |  |  |  |  |  |  |
| Exonic | 8,917,222 | 20,588 | 2.31 | 0.90 | 0.95 | -0.30 |
| Intronic | 552,778 | 1,634 | 2.96 | 1.14 | 1.21 | -0.61 |
| Intergenic | 2,964,129 | 7,050 | 2.38 | 1.04 | 1.09 | -0.48 |
|  |  |  |  |  |  |  |
| Low Complexity Exonic | 1,705,666 | 7,878 | 4.62 | 2.07 | 2.15 | -0.28 |
| Exonic excluding Low Complexity | 7,211,556 | 12,670 | 1.76 | 0.61 | 0.62 | -0.32 |
|  |  |  |  |  |  |  |
| Nonsyn Exonic | 6,003,434 | 12,475 | 2.08 | 0.82 | 0.84 | -0.27 |
| FFD  Exonic | 593,344 | 1,764 | 2.97 | 1.13 | 1.21 | -0.31 |
