# Supplementary material for: Long- and Short-Term Selective Forces on Malaria Parasite Genomes
Source: PLoS Genet. 2010 Sep 9;6(9):e1001099. doi: 10.1371/journal.pgen.1001099 (PMC2936524; doi:10.1371/journal.pgen.1001099)
Supplement: Table S7 — Mann-Whitney Tests comparing derived allele frequency (DAF) and minor allele frequency in different annotations of the genome. A. All genes. Tests are one-sided Mann-Whitney U Tests of MAF/DAF in annotation 1 against annotation 2. All tests are shown, sorted by P-value. The Bonferonni P-value = 0.05/12 = 0.0041667. Tests with P-values less than this Bonferonni-adjusted P-value are shown in bold. B. Excluding Var, Rifin and Stevor genes. All combinations of tests were performed, as above (exon vs intron, exon vs intergenic, DAF/MAF, greater/less). Only tests with P values<0.05 are shown. Tests with P-values less than the Bonferonni-adjusted P-value of 0.0041667 are shown in bold. C. Var, Rifin and Stevor genes only. All combinations of tests were performed, as above (exon vs intron, exon vs intergenic, DAF/MAF, greater/less). Only tests with P values<0.05 are shown. (0.06 MB DOC) [file pgen.1001099.s012.doc]

**Table S7. Mann-Whitney Tests comparing derived allele frequency (DAF) and minor allele frequency in different annotations of the genome.**

**Table S7a. All genes**

Tests are one-sided Mann-Whitney U Tests of MAF/DAF in annotation 1 against annotation 2. All tests are shown, sorted by P-value. The Bonferonni P-value = 0.05/12 = 0.0041667. Tests with P-values less than this Bonferonni-adjusted P-value are shown in bold.

| **Annotation 1** | **Annotation2** | **DAF/MAF** | **test alternative** | **P-value** | **Passes Bonf.** | **Indicates** |
| --- | --- | --- | --- | --- | --- | --- |
| **exon** | **intergenic** | **MAF** | **less** | **0.000145168** | **Y** | **exon MAF < intergenic MAF** |
| nonsyn | FFD | DAF | greater | 0.007745653 | N | nonsyn DAF > FFD DAF |
| exon | intron | DAF | greater | 0.05788475 | N |  |
| nonsyn | FFD | MAF | greater | 0.06521135 | N |  |
| exon | intron | MAF | less | 0.3861832 | N |  |
| exon | intergenic | DAF | greater | 0.4399369 | N |  |
| exon | intergenic | DAF | less | 0.560064 | N |  |
| exon | intron | MAF | greater | 6.14E-01 | N |  |
| nonsyn | FFD | MAF | less | 0.934789 | N |  |
| exon | intron | DAF | less | 0.9421158 | N |  |
| nonsyn | FFD | DAF | less | 0.9922545 | N |  |
| exon | intergenic | MAF | greater | 1.00E+00 | N |  |

**Table S7b. Excluding Var, Rifin and Stevor Genes**

All combinations of tests were performed, as above (exon vs intron, exon vs intergenic, DAF/MAF, greater/less).

Only tests with P values < 0.05 are shown. Tests with P-values less than the Bonferonni-adjusted P-value of 0.0041667 are shown in bold.

| **Annotation 1** | **Annotation2** | **DAF/MAF** | **test alternative** | **P-value** | **Passes Bonf.** | **Indicates** |
| --- | --- | --- | --- | --- | --- | --- |
| **exon** | **intergenic** | **MAF** | **less** | **1.79E-10** | **Y** | **exon MAF < intergenic MAF** |
| **nonsyn** | **FFD** | **DAF** | **greater** | **0.003115553** | **Y** | **nonsyn DAF > FFD DAF** |
| exon | intron | MAF | less | 1.42E-02 | N | exon MAF < intron MAF |
| nonsyn | FFD | MAF | greater | 0.04736657 | N | Nonsyn MAF > FFD MAF |

**Table S7c. Var, Rifin and Stevor Genes Only**

All combinations of tests were performed, as above (exon vs intron, exon vs intergenic, DAF/MAF, greater/less).

Only tests with P values < 0.05 are shown.

| **Annotation 1** | **Annotation2** | **DAF/MAF** | **test alternative** | **P-value** | **Passes Bonf.** | **Indicates** |
| --- | --- | --- | --- | --- | --- | --- |
| **exon** | **intergenic** | **MAF** | **greater** | **7.38E-10** | **Y** | **exon MAF > intergenic MAF** |
| **exon** | **intron** | **DAF** | **greater** | **0.000170436** | **Y** | **exon DAF > intron DAF** |
| **exon** | **intron** | **MAF** | **greater** | **0.000550602** | **Y** | **exon MAF > intron MAF** |
| exon | intergenic | DAF | greater | 0.01094373 | N | exon DAF > intergenic DAF |
