# Supplementary material for: Long- and Short-Term Selective Forces on Malaria Parasite Genomes
Source: PLoS Genet. 2010 Sep 9;6(9):e1001099. doi: 10.1371/journal.pgen.1001099 (PMC2936524; doi:10.1371/journal.pgen.1001099)
Supplement: Table S8 — GO-Slim Groups with an excess high frequency alleles. For all 23 GO categories, we compared the DAF of all SNPs within genes the GO-Slim category to all other SNPs using a two-tailed Mann-Whitney U test. We repeated the same analysis using minor allele frequencies. Since we conducted 46 tests the Bonferonni-corrected threshold is . Only categories with P values <0.05 are shown. (0.05 MB DOC) [file pgen.1001099.s013.doc]

**Table S8. GO-Slim Groups with an excess high frequency alleles**

For all 23 GO categories, we compared the DAF of all SNPs within genes the GO-Slim category to all other SNPs using a two-tailed Mann-Whitney U test. We repeated the same analysis using minor allele frequencies. Since we conducted 46 tests the Bonferonni-corrected threshold is 0.05/46 = 1.09 x10^-3^. Only categories with P values < 0.05 are shown.

| **Categories with an excess of high frequency alleles** | | | |
| --- | --- | --- | --- |
| **DAF/MAF** | **GO** | **GO description** | **P-value** |
| MAF | GO:0007154 | Cell communication | < 10^-16^ |
| MAF | GO:0044406 | Adhesion to host | < 10^-16^ |
| MAF | GO:0044413 | Avoidance of host defenses | < 10^-16^ |
| MAF | GO:0030260 | Entry into host cell | 1.13 x10^-2^ |
| MAF | GO:0007049 | Cell cycle | 1.25 x10^-2^ |
| MAF | GO:0007165 | Signal transduction | 1.96 x10^-2^ |
| **Categories with an excess of rare alleles** | | | |
| MAF | GO:0006139 | Nucleobase, nucleoside, nucleotide and nucleic acid metabolism | 5.21 x10^-6^ |
| MAF | GO:0051244 | Regulation of cellular physiological process | 5.87 x10^-6^ |
| DAF | GO:0006519 | Amino acid and derivative metabolism | 2.22 x10^-4^ |
| MAF | GO:0016043 | Cell organization and biogenesis | 2.10 x10^-3^ |
| DAF | GO:0016043 | Cell organization and biogenesis | 2.40 x10^-3^ |
| DAF | GO:0006139 | Nucleobase, nucleoside, nucleotide and nucleic acid metabolism | 5.15 x10^-3^ |
| DAF | GO:0019538 | Protein metabolism | 7.58 x10^-3^ |
| MAF | GO:0006519 | Amino acid and derivative metabolism | 8.76 x10^-3^ |
| DAF | GO:0044237 | Cellular metabolism | 2.85 x10^-2^ |
| MAF | GO:0005975 | Carbohydrate metabolism | 3.12 x10^-2^ |
