# Supplementary material for: Long- and Short-Term Selective Forces on Malaria Parasite Genomes
Source: PLoS Genet. 2010 Sep 9;6(9):e1001099. doi: 10.1371/journal.pgen.1001099 (PMC2936524; doi:10.1371/journal.pgen.1001099)

**Supplementary Text for:**

**Long and short term selective forces on malaria parasite genomes.**

Sanne Nygaard^2,3^, Alexander Braunstein^4,5^, Gareth Malsen^1^, Stijn Van Dongen^6^, Paul P. Gardner^1^, Anders Krogh^2^, Thomas D. Otto^1^, Arnab Pain^1^, Matthew Berriman^1^, Jon McAuliffe^4^, Emmanouil T. Dermitzakis^1,7*^, Daniel C. Jeffares^1,8*^.

**Supplementary Text**

**Estimating the number of constrained nucleotides in the *P. falciparum* genome**

To quantify constraint in more tangible terms, we can estimate the *number* of nucleotides in the *P. falciparum* genome that have been subject to constraint over long periods of time. We calculated the number of sites that were aligned in *P. falciparum* and at least three other species (‘well aligned sites’). These sites will include at least two of the main clades (Fig 1.), and will therefore contribute the majority of the signal of constraint. There are 10,160 kb well aligned *P. falciparum* nucleotides in the alignment, the majority of which are exonic (6,751 kb). Multiplying constraint by the number of well aligned sites provides a lower bound for the number of constrained nucleotides in each annotation. This is a lower bound, because there will certainly be sites that are constrained in only two or three species. This approach indicates that there are at least 5,982 kb constrained sites in the *P. falciparum* genome. This includes 4,765 kb constrained protein-coding exonic sites and 1,325 kb of constrained intergenic sites (Fig 2C). There are similar amounts of constrained sites in other species, e.g. *P. knowlesi* (Table S7). This analysis shows that while the majority of constrained (functional) sites in these genomes are protein-coding (~4,700 kb in *P. falciparum*), there are many constrained non-protein-coding sites in these genomes (mainly intergenic, ~1,300 kb in *P. falciparum* ).

**Conserved Elements are subject to purifying selection in P. falciparum and are not cold-spots**

A lack of divergence in Conserved Elements could, in principle, be due to ‘cold-spots’ for mutation events, rather than purifying selection. Polymorphism data can distinguish these possibilities because under purifying selection we expect an excess of rare alleles, a feature not expected in mutation ‘cold spots’ [1]. We used the *P. falciparum* SNPs we identified (see Methods) to test this. SNPs in CEs showed significant shifts to rare allele frequencies compared to similarly annotated non-CE SNPs (one sided Mann-Whitney tests of MAF distributions in CEs *vs.* non-CE regions, all sites P = 3.6x10^-97^, exonic P = 3.4x10^-62^, intergenic P = 1.41x10^-3^, intronic P = 0.2 (not significant)). This confirms that the predicted conserved elements (CEs) are subject purifying selection.

**Analysis of the proportion of multiple-of-three-spaced substitutions in conserved elements**

To quantify the synonymous substitution bias, we calculated the proportion of multiple-of-three-spaced substitutions (PMOT) for all 670 non-exonic CEs and a set of 670 exonic CE controls with the same length distribution. Non-exonic CEs had lower PMOT scores than exonic CEs (P < 2.2 x10^-16^), indicating that they are enriched for elements that do not encode proteins (Fig. 4b).

We found that 206 (31%) of the non-exonic CEs had PMOT scores less than the 5^th^ percentile of exonic CEs, these elements are very unlikely to be protein-coding exons. To determine if there are any elements that are very likely to be exons, we created a null distribution for the PMOT metric using shuffled alignments (since shuffling should remove any signal of multiple-of-three biased substitutions). We found that only 41 of the 670 (4.3%) non-exonic CEs had PMOT scores above the 95^th^ percentile of the null distribution, slightly fewer than the 5% we would expect at random, indicating that very few are protein-coding.

**Analysis of the similarity of CEs to the genomes of related Alveolates using tblastx.**

Protein-coding elements are also expected to be conserved at the amino-acid sequence level between related species. Plasmodium parasites are related to a group of unicellular eukaryotes called Alveolates. To quantify this conservation, we performed a translated BLAST search (tBLASTx) of CE sequences against 10 Alveolate genomes (Table S8), recording the average BLAST bit score for each of the 670 non-exonic CEs and 670 exonic CE controls (Fig 4c, see methods). In support the of PMOT metric, exonic elements had significantly higher BLAST scores than non-exonic elements (Mann-Whitney test P = 3.7 x10^-3^). We found that 188 of the 670 (28%) non-exonic elements had BLAST scores less than the 5^th^ percentile of exonic control elements, these elements are very unlikely to be protein-coding.

**Examining how many conserved elements might encode ncRNAs**

It is possible that some of the CEs we discover have been conserved because they encode a structured, non-protein-coding RNA (ncRNA). This includes those that overlap exon annotations, since a recent experimental study [2] found 328 short antisense RNAs that overlapped exons in *P. falciparum.* Only 239 of the 16,649 CEs in *P. falciparum* (1.4 %) overlap previously discovered ncRNAs [2,3], including exonic antisense ncRNAs. However it is possible that there are many more, as yet undiscovered ncRNAs in these genomes. To examine this possibility, we used the RNAz algorithm [4] which predicts whether aligned sequences could form a conserved secondary structure. We examined both exonic and non-exonic CEs because in principle mRNA transcripts could well encode RNA structures.

RNAz is known to have high false positive rates [5], so we generated one simulated alignment for each CE using SISSIz (Version 0.1) [5]. SISSIz produces simulated multiple alignments from an input alignment preserving dinucleotide content, sequence diversity, local conservation and gap patterns. We then used RNAz to produce a support vector probability (SVP) for each real (native) CE alignment and each simulated CE alignment.

There were 8,726 exonic CEs and 95 non-exonic CEs where we could produce an RNAz prediction from both the native and the simulated alignment. Both exonic CEs and non-exonic CEs showed significantly higher SVPs than the simulated alignments (Mann-Whitney tests, P < 2.2x10^-16^ and P = 1.70x10^-7^), indicating that they contain genuine structural RNAs. Using the conservative and taking the false discovery rate into account, we estimate that 644 of the exonic and 22 non-exonic CEs encode ncRNAs with secondary structure (see Methods). Since only 239 of our CEs overlap known ncRNAs, this suggests that there are many more structured RNAs that remain to be discovered, particularly within protein-coding exons.

**Expression Levels of Conserved Elements in P. falciparum**

We examined transcription levels within CE’s using data from a recent RNA-Seq analysis of *P. falciparum* red blood cell stage parasites [6]. In *P. falciparum* there are 16,648 CEs (≥ 25nt length). Of these 16,121 (97%) overlap exon annotations. Of the 527 non-exonic elements, 71 (13%) are likely to be non-coding RNAs, because they overlap ncRNAs annotated in the genome, predicted [3], or experimentally defined [2]. The remaining 456 are not annotated (87 %). RNA-Seq data showed that both the exonic CEs and likely ncRNA set are transcribed at higher levels than the un-annotated set (Mann-Whitney tests, P < 2.2x10^-16^ and P = 1.6 x10^-16^), supporting these annotations.

Overall, we found 46% of the non-exonic CEs (244/433) were transcribed at a plausibly biologically meaningful level (the 5^th^ percentile of exon CEs). However, there is considerable non-exonic transcription in *P. falciparum* [6]. To examines whether intergenic CEs were particularly enriched for transcription (compared to other intergenic regions) we compared their RNAseq expression levels to a set of length and GC-content matched control intergenic control regions (see Methods). There was no significant difference between the intergenic CE’s and controls (P > 0.05), nor between intronic CE’s and similarly matched intronic controls. This further supports our conclusion that not many of the CEs we discover are ncRNAs are exons, or long structured ncRNAs. It should be noted that the methods used by Otto et al. would not detect selection in short (< 200nt) ncRNAs, nor exons or ncRNAs that are expressed in developmental stages other than the intra-erythrocytic cycle.

**Purifying selection within *P. falciparum* (using derived allele frequencies) correlates with long term constraint (using GERP).**

We expected that that the degree of purifying selection within *P. falciparum* will be correlated with the long-term selective constraint that we observe in the seven Plasmodium species alignment. We expect this to hold throughout the genome, including intergenic regions. The derived allele frequency of SNPs (DAF) is a suitable proxy for constraint within a population because it will be lower when constraint is high [7,8]. We calculated the median number of ‘rejected substitutions’ estimated by GERP from the seven species alignment in the 5nt window around each SNP, as a proxy to constraint at that site in the genome. The DAF and the GERP constraint measure were negatively correlated (Pearson's correlation r = -0.102, P = 1.02 x10^-33^), indicating that regions that are conserved over long periods of time have generally remained subject to stronger purifying selection in *P. falciparum*. This observation is also significant within only exonic or intergenic regions, but not intronic sites (exonic SNP DAF and median GERP score r = -0.14, P < 2.2x10^-16^, intergenic SNPs r = -0.05, P = 1.6x10^-3^, intronic r = -0.04, P = 0.08).

**References**

1. Drake JA, Bird C, Nemesh J, Thomas DJ, Newton-Cheh C, et al. (2006) Conserved noncoding sequences are selectively constrained and not mutation cold spots. Nat Genet 38: 223-227.

2. Raabe CA, Sanchez CP, Randau G, Robeck T, Skryabin BV, et al. (2009) A global view of the nonprotein-coding transcriptome in Plasmodium falciparum. Nucleic Acids Res.

3. Mourier T, Carret C, Kyes S, Christodoulou Z, Gardner PP, et al. (2008) Genome-wide discovery and verification of novel structured RNAs in Plasmodium falciparum. Genome Res 18: 281-292.

4. Washietl S, Hofacker IL, Stadler PF (2005) Fast and reliable prediction of noncoding RNAs. Proc Natl Acad Sci U S A 102: 2454-2459.

5. Gesell T, Washietl S (2008) Dinucleotide controlled null models for comparative RNA gene prediction. BMC Bioinformatics 9: 248.

6. Otto TD, Wilinski D, Assefa S, Keane TM, Sarry LR, et al. (2010) New insights into the blood-stage transcriptome of Plasmodium falciparum using RNA-Seq. Mol Microbiol 76: 12-24.

7. Nielsen R (2005) Molecular signatures of natural selection. Annual Review of Genetics 39: 197-218.

8. Andolfatto P (2005) Adaptive evolution of non-coding DNA in Drosophila. Nature 437: 1149-1152.

**Manual Examination of Non-exonic CE’s In *P. falciparum***

We selected 20 *P. falciparum* non-exonic CE’s from chromosome 14, by selecting every 4^th^ CE from the 79 non-exonic CE’s present on this chromosome.

We examined the genomic context of these 67 elements in more detail using the Artemis annotation tool ([www.sanger.ac.uk/Software/Artemis/](http://www.sanger.ac.uk/Software/Artemis/)), which allowed us to view annotations, RNA-Seq expression, AT content, start and stop codons etc. Of these 20 elements, our best appriasal of their functions were:

exon: 5 elements (25%)

ncRNA: 2 elements (10%)

genuine functional element, but function unknown: 8 elements (40%)

elements whose functional status looks equivocal, possible artifactual alignment: 4 (20%)

Details and comments below. In Artemis screen shots CE’s are shown as white boxes named ‘GCE’ (GERP Conserved Element).

--------------------------------------------------------------------------------

Id: GCE.1.74740.74766

Pf chr: MAL14

Pf start: 119600

Pf end: 119626

Annotation: Pf_intron Pk_intergenic Py_intergenic

Av_blast_bit: 29.414

Av_blast_eval: 3.61428571428571

PMOT: 0.394003527336861

Percent_AT: 0.877

GerpPvalue: 1.16954e-06

Gene: PF14_0031

Len: 27

Rnaseq_gm: 1

Rnaz_prob: NA

CLUSTAL W(1.81) multiple sequence alignment

PlaBer/40542-40566 GAAAAAAAAGAGGAATAAAAAAAAG--

PlaYoe/45225-45251 GAAAAAAAAGAGGAATAAAAAGAGGAA

PlaCha/21644-21666 AAAAAAAAAGAGGAATAAAAAAA----

PlaViv/60202-60225 GGAAAAAAAAAAAAAAAAAAAAGA---

PlaKno/60638-60664 GAAAAAAAAAAAAAAAAGAAAAAAAAA

PlaFal/49233-49259 GAAAAAAAAAAAAAAAAAAAAAAAAAA

PlaRei/49233-49259 GAAAAAAAAAAAAAAAAAAAAAAAAAA

******* * ** * ***

Notes: Intron of P. falciparum, very low/no expression, in repeat.

Function: equivocal.

--------------------------------------------------------------------------------

Id: GCE.1.96248.96276

Pf chr: MAL14

Pf start: 132072

Pf end: 132100

Annotation: Pf_intergenic Pk_intergenic Py_intergenic

Av_blast_bit: 32.140

Av_blast_eval: 0.976

PMOT: 0.102564102564103

Percent_AT: 0.660

GerpPvalue: 2.40313e-08

Gene: PF14_0810 PF14_0033

Len: 29

Rnaseq_gm: 6.68

Rnaz_prob: NA

CLUSTAL W(1.81) multiple sequence alignment

PlaBer/53189-53217 TCTTTTACAATTTTGTAATTTTTTTCCAT

PlaYoe/58444-58472 TCTTTTACAATTTTGTAATTTTTTTCCAT

PlaViv/73825-73853 TTTTTTACAATGTTGTGATTCTTTTCCAT

PlaKno/74362-74390 TTTTTTACAATGTTGTGATTCTTTTCCAT

PlaFal/61705-61733 TTTTTTACTATTTGGTGATTTTTTTCCAT

* ****** ** * ** *** ********

Notes: Now annotated as exon of PF14_0810, from RNA-Seq data. See fig.

Function: exon


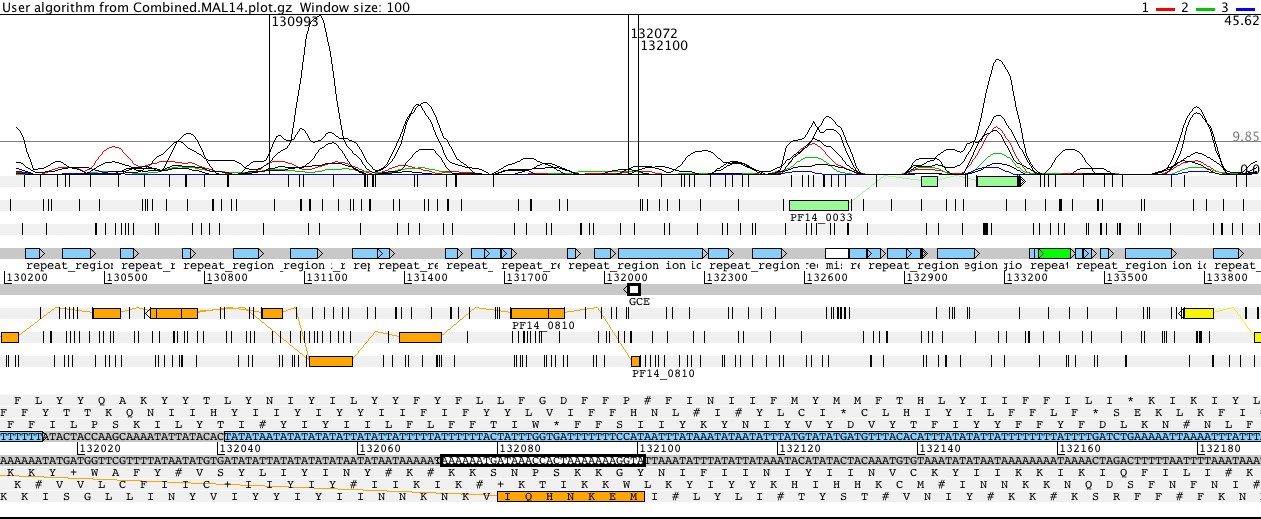


**Figures:** Artemis screen shots show RNA-Seq expression at top (from Otto et al. 2010, colours indicate expression in multiple developmental time points of erthrocytic cell cycle), gene annotations in middle (vertical bars indicate stop codons), and sequence (with 6 frame translation) below. Gene annotation includes changes made due to RNA-Seq data and annotation jamboree (2008).

--------------------------------------------------------------------------------

Id: GCE.1.201714.201741

Pf chr: MAL14

Pf start: 199108

Pf end: 199135

Annotation: Pf_intergenic Pk_intergenic

Av_blast_bit: 27.650

Av_blast_eval: 3.6

PMOT: 0

Percent_AT: 0.802

GerpPvalue: 2.78808e-05

Gene: PF14_0054 PF14_0055

Len: 28

Rnaseq_gm: 10.41

Rnaz_prob: NA

CLUSTAL W(1.81) multiple sequence alignment

PlaBer/91133-91134 --------------------------CT

PlaYoe/111476-111503 NNNNNNNNNNNNNNNNNNNNNNNNNNNT

PlaViv/148137-148164 TTTTTTTTTTTTTTTTTTTTTTTTTTTT

PlaKno/146957-146984 TTTTTTTTTTTTTTTTTTTTTTTTTTTT

PlaFal/128741-128768 TTTTTTTTTTTTTTTTTTTTTTTTTTTT

PlaRei/128741-128768 TTTTTTTTTTTTTTTTTTTTTTTTTTTT

*

Notes: Entirely T sequence. In repeat.

Function: equivocal.

--------------------------------------------------------------------------------

Id: GCE.1.563646.563794

Pf chr: MAL14

Pf start: 429285

Pf end: 429411

Annotation: Pf_intergenic Pk_intergenic Py_intergenic

Av_blast_bit: 34.417

Av_blast_eval: 1.08476666666667

PMOT: 0.125110158696364

Percent_AT: 0.784

GerpPvalue: 6.19543e-05

Gene: PF14_0104 MAL14_17_candidate_ncRNA

Len: 149

Rnaseq_gm: 2.55

Rnaz_prob: 0.132503

CLUSTAL W(1.81) multiple sequence alignment

PlaBer/207085-207214 AAAAATGCAAATATAATA---------TATATGCATACAAAACATA--------ATT--T

PlaYoe/288253-288382 AAAAATGCAAATATAATA---------TATATGCATACAAAACATA--------ATT--T

PlaCha/139685-139814 AAAAATGCAAATATAATA---------TATATGCATACAAAACATA--------ATT--T

PlaViv/394195-394334 AAAAAATAAAAAAAAAAAGGAATAGCCTAAACGTATATGTAAAATCGCACTCGCATTCCT

PlaKno/385270-385407 -----ATAAAACGAAAAAGGATTAGCCTACATGTATAAGTAAAATCACATTTGCATTCCT

PlaFal/358918-359044 AAAAAATAGAAAAAAAAAAAAATAAAACTAATAAGGAGAAAAAGGAAAAATAATAATCCT

PlaRei/358918-359044 AAAAAATAGAAAAAAAAAAAAATAAAACTAATAAGGAGAAAAAGGAAAAATAATAATCCT

** ** * * * ** * * *

PlaBer/207085-207214 TTAAAAATTGATTCTTGCCCACGAAACATACATAGGACAAAGGATACAGTAAATACATGT

PlaYoe/288253-288382 TTGAAAATTAATTCTTGCCCACGAAACATACATAGGGCAAAGAATACAGTAAATACATGT

PlaCha/139685-139814 TTGAAAATTAATTCTTGCCCACGAAATATACATAGGACAAAAAATACAATAAATACATGT

PlaViv/394195-394334 TTAAAAATTAACACATTTAACACAAACATAGGTGGGGCAAA------AG---GTCCGTAA

PlaKno/385270-385407 TCAAAAATTAACACATTTAACACAAACATAGATGGAGCAAA------AGAAGGCCCATAA

PlaFal/358918-359044 TTATTAATTTATATATGTAATTT-TATATGAATATGTTAAAAAAAAAAAAAAAAAGGAAA

PlaRei/358918-359044 TTATTAATTTATATATGTAATTT-TATATGAATTTGTTAAAAAAAAAAAAAAAAAGGAAA

* **** * * * ** * *** *

PlaBer/207085-207214 ACATATAACTTAAATACTTTTTGAATTCA

PlaYoe/288253-288382 ACATATAACTTAAATACTTTTTGAATTCA

PlaCha/139685-139814 ACATATAACTTAAATACTTTTTGAATTCA

PlaViv/394195-394334 ACTGTAAAGGTGGAGGGCTTTTTAATTCA

PlaKno/385270-385407 ATAGTAAAGGTGAAGAACTTTTTAATTCA

PlaFal/358918-359044 AAAAAAAA---------------------

PlaRei/358918-359044 AAAAAAAA---------------------

* **

Notes: Very low transcription in Pf. Alignment convincing.

Function: unknown

--------------------------------------------------------------------------------

Id: GCE.1.612694.612746

Pf chr: MAL14

Pf start: 460904

Pf end: 460956

Annotation: Pf_intergenic Pk_intergenic Py_intergenic

Av_blast_bit: 32.760

Av_blast_eval: 0.3382

PMOT: 0.33453548085901

Percent_AT: 0.518

GerpPvalue: 5.4969e-09

Gene: PF14_0111 PF14_0112

Len: 53

Rnaseq_gm: 38.77

Rnaz_prob: 0.000002

CLUSTAL W(1.81) multiple sequence alignment

PlaBer/229244-229296 GCGCGGAATTGATTTTTCAGGCTCTAAACAATGCGTTCAAAGAAAAAAAGGGT

PlaYoe/318132-318184 GCGCGGAATTAATTTTTCAGGCTCTAAACAATGCGTTCAAAGAAAAAAAGGGT

PlaViv/428412-428464 GAGCGGAGATGATCTTCCAAGCGTTGAACAATGCCTACAAGGAAAAAAAAAAC

PlaKno/419068-419120 GAGCAGAGATGATCTTCCAAGCGTTAAACAATGCCTACAAGGAAAAAAAAAAC

PlaFal/390537-390589 GAGCAGAAATGATTTTCCAAGCCTTAAATAACTCCTATAAGGATAAAAGGAAT

* ** ** * ** ** ** ** * ** ** * * ** ** ****

Notes: Now annotated as exon of PF14_0111, not PMOT high blast Eval low. Short exon.

Function: exon

--------------------------------------------------------------------------------

Id: GCE.1.679557.679769

Pf chr: MAL14

Pf start: 509171

Pf end: 509342

Annotation: Pf_intergenic Pk_intergenic Py_intergenic

Av_blast_bit: 33.129

Av_blast_eval: 1.00558571428571

PMOT: 0.151919645952452

Percent_AT: 0.760

GerpPvalue: 4.15277e-10

Gene: PF14_0124 PF14_0125

Len: 213

Rnaseq_gm: 33.00

Rnaz_prob: 0.025227

CLUSTAL W(1.81) multiple sequence alignment

PlaBer/266433-266606 GTATATGCACACC-----------TATTCGTTTTGCTATATACTTTTCTTCGTAGCTATT

PlaYoe/357140-357313 GTATATGCACACC-----------TATGCGTTTTGCTATATACTTTTCTTCGTAGCTATT

PlaCha/171650-171818 ATATATGCACACC-----------TATGCGTCTTTCTACGTACTTTTCTTCGTAGC----

PlaViv/479083-479281 GTCCATGCATAC-AGGCAACCC-----CCCCTTT------CGCTCTGCCAAGTCTCCATT

PlaKno/469413-469617 GTCCATGCATACCAGACAACCCTTCACCCCTTTT------TGCTCTGCCAAGTCTCCATT

PlaFal/438804-438975 ATATTTGCTTAATAATTAAATATTTCCCCCCCATCCCACATTACATAATATATATACATA

PlaRei/438804-438975 ATATTTGCTTAATAATTAAATATTTCCCCCCCATCCCACATTACATAATATATATACATA

* *** * * * * *

PlaBer/266433-266606 TCATT--TATTTTATTTGACATTCACAAAAT--TATTCTTATA-----AAAAATTCATTA

PlaYoe/357140-357313 TCATT--TATTTTATTTGACATTCACAAAAT--TATTCTTATA-----AAAAATTCATTA

PlaCha/171650-171818 -------TATTTTATTTGACATTCACAAAAT--TATTCTTATACTTATAAAAAATCATTA

PlaViv/479083-479281 TGAATCCTATTTAGTTTGACATTCCCCAAATAATATTATGATCTTTATTTTCATTTTTGA

PlaKno/469413-469617 TGAATCCTACGTAGTTTGAAATTCCTCAAATAATATTATCATATTTATTTTCATTTTTGA

PlaFal/438804-438975 T--------------------------ATATAATATGACCACGTTTTTTCTGAATAATTA

PlaRei/438804-438975 T--------------------------ATATAATATGACCACGTTTTTTCTGAATAATTA

* ** *** * * * * *

PlaBer/266433-266606 AA----------------A-TATACATTTTTCTAATTCCTAAAATATATTTACTTTTAAA

PlaYoe/357140-357313 AA----------------A-TATACATTTTTATAATTCCTGAAATATATTTGCTTTTAAA

PlaCha/171650-171818 AA----------------A-TATACATTTTGCTAATTCCTGAAATATATTTGCTTTTAAA

PlaViv/479083-479281 AA-CGTATTTGTTTAAAGA-TTAAATTTTTGGTGATTCTCTAAGTGCATTTGTTTTAGCA

PlaKno/469413-469617 AA-CGTATTTGTTTAAAGA-TTAAATTTTTGGTGATTCTCTAAGTGCATTTGTTTTAGCA

PlaFal/438804-438975 AATCTTATTTGTTTAAAGACTAAAATTTTTGGTAATTTATAAAATGAATTTGTTTTACCA

PlaRei/438804-438975 AATCTTATTTGTTTANAGACTAAAATTTTTGGTAATTTATAATATGAATTTGTTTTNCCA

** * * * **** * *** * * **** *** *

PlaBer/266433-266606 TGGACAATTTTTTT--AAGAGGAATTTGTTATT

PlaYoe/357140-357313 TGCACAATTGTTTT--AAGAGGAATTTGTTATT

PlaCha/171650-171818 TGCACAATTGTTTT--AAGAGGAATTTGTCA-T

PlaViv/479083-479281 TATTCCATTTTTTTTAAAAAGCAGTTTGTTATT

PlaKno/469413-469617 TATTTCATTTTTTTTTAAAATCAGTTTGTTATT

PlaFal/438804-438975 TATAATATATATATATA---------------T

PlaRei/438804-438975 TATAATATACATATATA---------------T

* ** * * * *

Notes: Strongly transcribed, but has many stop codons in all frames. Present in all seven species. Trsnscriptino continuous with gene PF14_0124. Could be UTR of either PF14_0124/PF14_0125. Low RNAz score. See fig.

Function: unknown, UTR?


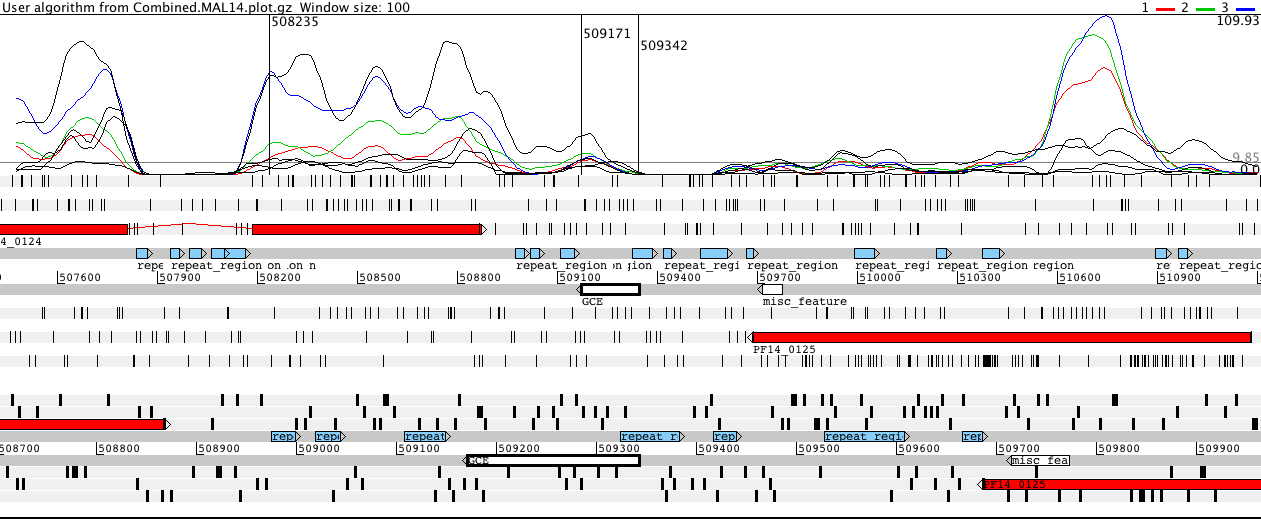


--------------------------------------------------------------------------------

Id: GCE.1.823423.823477

Pf chr: MAL14

Pf start: 577757

Pf end: 577804

Annotation: Pf_intergenic Pk_intergenic Py_intergenic

Av_blast_bit: 31.617

Av_blast_eval: 1.751

PMOT: 0.188265306122449

Percent_AT: 0.793

GerpPvalue: 2.04641e-10

Gene: PF14_0142 PF14_0143

Len: 55

Rnaseq_gm: 7.48

Rnaz_prob: NA

CLUSTAL W(1.81) multiple sequence alignment

PlaBer/299588-299638 CTTATATTTAAT-TTTTTTTAATATGAATT--ATTTATGTA-TTATTATAAAAAC

PlaYoe/402854-402903 CTTATATTTAA--TTTTTTTAATATGAATT--ATTTATGTA-TTATTATAAAAAC

PlaCha/210170-210221 CTTATATTTAATTTTTTTTTAATATGAATT--ATTTATGTA-TTATTATAAAAAC

PlaViv/556525-556574 CTTATATTTAAA---TTATTAATATGAATT--ATTCATGTATTTATTAAAAAAAA

PlaKno/576424-576472 CTTATATTTAAA---TTATTAATATGAATT--ATTCATGTACTTAAT-AAAAAAA

PlaFal/507390-507437 ----TATTTAAA---TTATTAATATGAATTTTATTTATGTATTTATTATAAAAAC

******* ** ************ *** ***** *** * *****

Notes: AT rich, strongly conserved in 6 species (P.reich missing), transcribed. One frame (4), is stop-codon free, encodes CFYNKYINKIHINNLNN, best NCBI nr blastp hit is hypothetical protein DDB_G0288975 [Dicty] E = 0.42. Perhaps last exon of gene either side. Annotated as CDS, but protein-coding staus equivocal. See fig.

Function: unknown


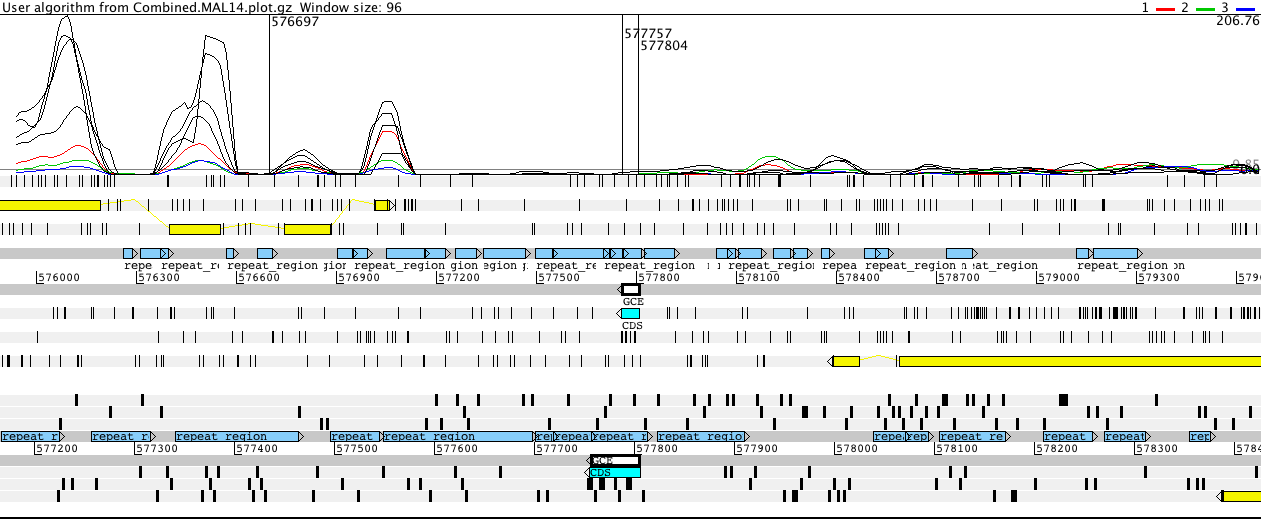


--------------------------------------------------------------------------------

Id: GCE.1.1333419.1333505

Pf chr: MAL14

Pf start: 875190

Pf end: 875274

Annotation: Pf_intergenic Pk_intergenic Py_intergenic

Av_blast_bit: 31.000

Av_blast_eval: 0.928666666666667

PMOT: 0.192063960474909

Percent_AT: 0.767

GerpPvalue: 1.60848e-08

Gene: PF14_0204 PF14_0205

Len: 87

Rnaseq_gm: 5.04

Rnaz_prob: 0.116620

CLUSTAL W(1.81) multiple sequence alignment

PlaBer/424687-424760 AAGAAGAAATCCCTTATTACAATTTTTTTAGAATTTGCATCAT-------------TTTT

PlaYoe/574972-575045 AAGAAGAAATCTCTTATTACAATTTTTTTAGAATTTGCATCAC-------------TTTT

PlaCha/310513-310584 AAGAAGAAATCTTTTA-TATGACTTTTTTAGAATTTGCATCAC--------------TGT

PlaViv/863947-864027 AAGAAGAAATCTTTTCAT--CACTTGTTCAAAA-TTGCGTCACCCTTTGTTGAGTTTTTC

PlaKno/853856-853939 AAGAAGAAATCTTTTATT--CACTTTTTCAAAA-TTGCGTCACCTTTTATTTAGTTTTTT

PlaFal/804823-804907 AAGAAGAAATCTTTTATTACATTTTTTTTAAGTTCCGCATCACCTTTTTTTATATTTTTT

PlaRei/804823-804907 NNNNNGAAATCTTTTATTACATTTTTTTTAAGTTCCGCATCACCTTTTTTTATATTTTTT

****** ** * ** ** * ** *** *

PlaBer/424687-424760 TTTGTTATCATCATTTTATTTCACTTA

PlaYoe/574972-575045 TTTGTTATCATCATTTTATTTCACTTA

PlaCha/310513-310584 TTTGTTATCATCATATTATTTCATTTA

PlaViv/863947-864027 TTTA---TCATCATGTTGTTTCGTCCA

PlaKno/853856-853939 TTTTGTGTCATCATTTTGTTCCATTCA

PlaFal/804823-804907 TTT--TATCATCACTTTATTTCATTTA

PlaRei/804823-804907 TTT--TATCATCACTTTATTTCATTTA

*** ****** ** ** * *

Notes: Weakly transcribed, stops in all but frame 2, could be last exon of gene PF14_0205, but much less transcribed. See fig.

Function: unknown


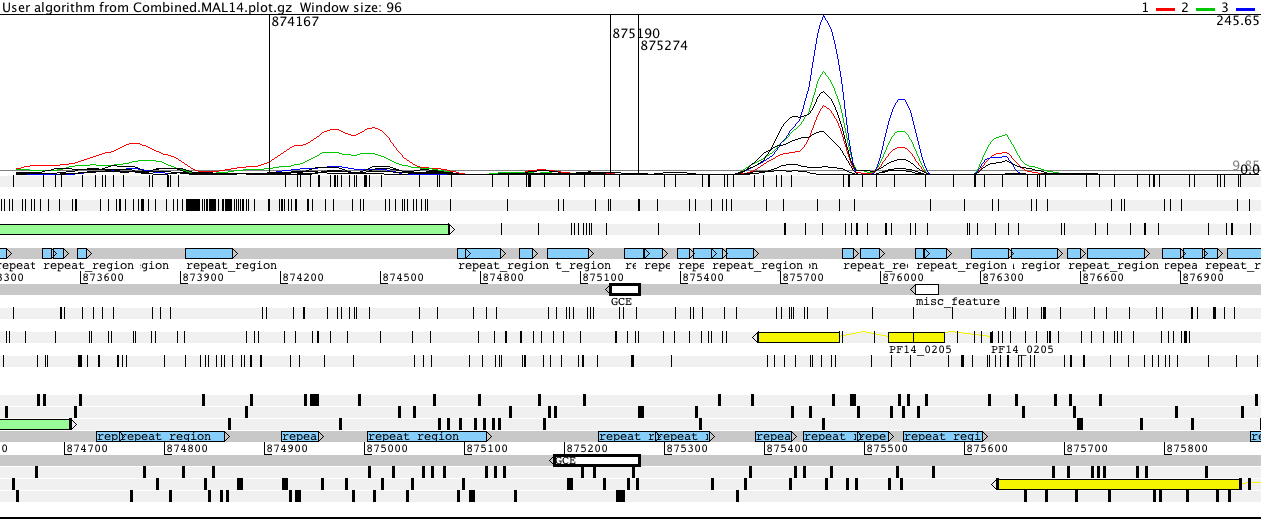


--------------------------------------------------------------------------------

Id: GCE.1.1496414.1496570

Pf chr: MAL14

Pf start: 975187

Pf end: 975332

Annotation: Pf_intron Pk_intron Py_intron

Av_blast_bit: 32.800

Av_blast_eval: 0.334285714285714

PMOT: 0.164412050783651

Percent_AT: 0.692

GerpPvalue: 1.42949e-10

Gene: PF14_0230

Len: 157

Rnaseq_gm: 27.49

Rnaz_prob: 0.612783

CLUSTAL W(1.81) multiple sequence alignment

PlaBer/474971-475119 TGTTCCAGTGCCAGGTGAGTCAAAAATGTTTATAAATTTTTTTTAACCCGTTTTATCACT

PlaYoe/643943-644083 TGTGCCAGTGTCAGGTGAGTCAAAAATGTTTGTAAA-TTTTTTTAACCCGTTTTATCACT

PlaCha/357967-358113 TGTGCCAGTGTCAGGTGGGTCAAAAATTTTCGTAAAA--CTTTTAACCCGTTTTATCACT

PlaViv/962259-962402 TGTGCCAAAGTCAGGTGAGTCAGCAACTTGCGGCGAATGC--GTAACTCGTTTTATCAGT

PlaKno/951858-952004 TGTGCCAAAGTCAGGTGAGTTAGCAACTTGCGGAAAATGC--GTAACTCGTTTTATCAGT

PlaFal/904820-904965 TGTTCCAATGTCAGGTGAGTCGGCAACTTAAAAAAAAATGCT-TAACTCGTTTTATCATT

PlaRei/904820-904965 TGTTCCAATGTCAGGTGAGTCGGCAACTTAAAAAAAAATGCT-TAACTCGTTTTATCATT

*** *** * ****** ** ** * * **** ********** *

PlaBer/474971-475119 GATCATAAAAATTT------ATTTAGCCT-ATATAAATATGCTCTCATAATATTAGCTAC

PlaYoe/643943-644083 G---ATAAAAATTT------ATTTAGCCT-ATATAAATATTCTCTCACAATATT-GCTAC

PlaCha/357967-358113 GATAAGAAAAATTT------ATTTAGCCT-ATATAAATATGCTCTCATAATATTAGCTAC

PlaViv/962259-962402 GGCATTTTT---TTTTCTCATTTTAGCCTCGAATAAAGCAACTCTCATAACTTGTGCTA-

PlaKno/951858-952004 GGCATTTTTGTTTTTTTTCAATTTAGCCTCGAATAAAACATCTCTCATAACATTGGCTA-

PlaFal/904820-904965 G-CATAAACATTTTTT-----TTTAGCCTCTAATAAAAACATCCTCATAACATT---TTT

PlaRei/904820-904965 G-CATAAACATTTTTT-----TTTAGCCTCTAATAAAAACATCCTCATAACATT---TTT

* ** ******** ***** **** ** * *

PlaBer/474971-475119 ATATCATGAATGTAT-TGGGCATTTATTTAAGGCGTA

PlaYoe/643943-644083 ATATCATGAATGTAT-TGGGTATTTATTTAAGGC---

PlaCha/357967-358113 ATATCATGATTGCAT-TTAGCATTTATTTAAGGCATA

PlaViv/962259-962402 ---TTAAGAACGCGT-T---CGTTTATTTAAGGCATG

PlaKno/951858-952004 ---TTAAGAACGTAT-T---CATTTATTTAAGGCATG

PlaFal/904820-904965 ACATCATGAATTATTGTTAACATTTA-TTAAGGCATA

PlaRei/904820-904965 ACATCATGAATTATTGTTAACATTTA-TTAAGGCATA

* * ** * * **** *******

Notes: High transcription, possibly due to location in intron of PF14_0230. Stops in all 6 frames.

Function: unknown.

--------------------------------------------------------------------------------

Id: GCE.1.1510738.1510819

Pf chr: MAL14

Pf start: 982379

Pf end: 982455

Annotation: Pf_intergenic Pk_intergenic

Av_blast_bit: 32.367

Av_blast_eval: 0.76

PMOT: 0.207928626928627

Percent_AT: 0.758

GerpPvalue: 1.5941e-05

Gene: MAL14_27_candidate_ncRNA MAL14_28_candidate_ncRNA_selected

Len: 82

Rnaseq_gm: 110.70

Rnaz_prob: 0.986575

CLUSTAL W(1.81) multiple sequence alignment

PlaBer/477665-477743 ATCTCATGTATATTT--TCATTTTATTTATAAGTTTTTTATAAATAAAATTATAACTATA

PlaYoe/650201-650279 ATCTCATGTATATTT--TCATTTTATTTATAAGTTTTTTATAAATAAAATAGTAACTATA

PlaViv/970367-970447 ATCCCACGTGTATT-ATCGCTTTACTTTACGAGTTATTCGTGAGTAAGAGGCGAGTTAAT

PlaKno/960860-960938 ATCCCACGTCTATTTATCGTTTTACTTTACGAGTTTT---TTCGTAAGAGACGAATTAAT

PlaFal/912012-912088 ATCACATGTGTATTTTT--ATTTTATTTATAAGTTTTTTATGAATAAAATTAG---TAGT

PlaRei/912012-912088 ATCACATGTGTATTTTT--ATTTTATTTATAAGTTTTTTATGAATAAAATTAG---TAGT

*** ** ** **** *** **** **** * * *** * **

PlaBer/477665-477743 AATATATATGTTTCATGCGAT-

PlaYoe/650201-650279 AATATATATGTTTCATGCGAT-

PlaViv/970367-970447 AATATGTGTGCTTCATGGGGTA

PlaKno/960860-960938 AATATTCGTGCTTCATGGGGTA

PlaFal/912012-912088 AATATACATGCTTCGTGCGATA

PlaRei/912012-912088 AATATACATGCTTCGTGCGATA

***** ** *** ** * *

Notes: Highly transcribed in Novel Transcript PF14TR004 defined from RNA_Seq data (Otto 2010), also predicted to be ncRNA by Mourier (2008), and has nigh RNAz SVP.

Function: ncRNA

--------------------------------------------------------------------------------

Id: GCE.1.1690806.1690833

Pf chr: MAL14

Pf start: 1101337

Pf end: 1101364

Annotation: Pf_intergenic Pk_intergenic

Av_blast_bit: 29.250

Av_blast_eval: 4.705

PMOT: 0

Percent_AT: 0.75

GerpPvalue: 2.92595e-05

Gene: PF14_0259 PF14_0260

Len: 28

Rnaseq_gm: 29.70

Rnaz_prob: NA

CLUSTAL W(1.81) multiple sequence alignment

PlaViv/1091046-1091073 AAAAAAAAAAAAAAAAAAAAAAAAAAAA

PlaKno/1060209-1060236 AAAAAAAAAAAAAAAAAAAAAAAAAAAA

PlaFal/1030970-1030997 AAAAAAAAAAAAAAAAAAAAAAAAAAAA

****************************

Notes: A-rich tract.

Function: equivocal

--------------------------------------------------------------------------------

Id: GCE.2.58022.58047

Pf chr: MAL14

Pf start: 1519335

Pf end: 1519360

Annotation: Pf_intergenic Pk_intergenic

Av_blast_bit: 29.125

Av_blast_eval: 4.05

PMOT: 0

Percent_AT: 0.885

GerpPvalue: 3.42156e-05

Gene: PF14_0354 PF14_0355

Len: 26

Rnaseq_gm: 3.88

Rnaz_prob: NA

CLUSTAL W(1.81) multiple sequence alignment

PlaCha/9410-9418 -----------------ATATATATA

PlaViv/28877-28902 TATATATATATATATATATATATATA

PlaFal/24582-24607 TACATATATATATATATATATATATA

PlaRei/24582-24607 TACATATATATATATATATATATATA

*********

Notes: Conserved TA repeat region.

Function: equivocal

--------------------------------------------------------------------------------

Id: GCE.5.68557.68581

Pf chr: MAL14

Pf start: 1668168

Pf end: 1668192

Annotation: Pf_intergenic Pk_intergenic Py_intergenic

Av_blast_bit: 33.900

Av_blast_eval: 0.1965

PMOT: 0.0910493827160494

Percent_AT: 0.577

GerpPvalue: 2.7755e-06

Gene: PF14_0387 PF14_0388

Len: 25

Rnaseq_gm: 2.04

Rnaz_prob: NA

CLUSTAL W(1.81) multiple sequence alignment

PlaBer/33145-33169 TTTTTTCTATGCATACATAGCCATA

PlaYoe/42922-42946 TTTTTTCTATGCATATATAGCCATA

PlaCha/17249-17273 TTTTTTCTATGCATACATAGCCATA

PlaViv/46446-46470 TTCCTCCCGTGCACATATAAGCACA

PlaKno/46514-46538 TTTCTCCCGTGCACATATAAGCATA

PlaFal/43761-43785 TTTTTTGTATGCACATATAGGCATA

** * **** * *** ** *

Notes: Short intergenic element. Could be exon of up or downstream gene, has several ORFs. See fig.

Function: unknown


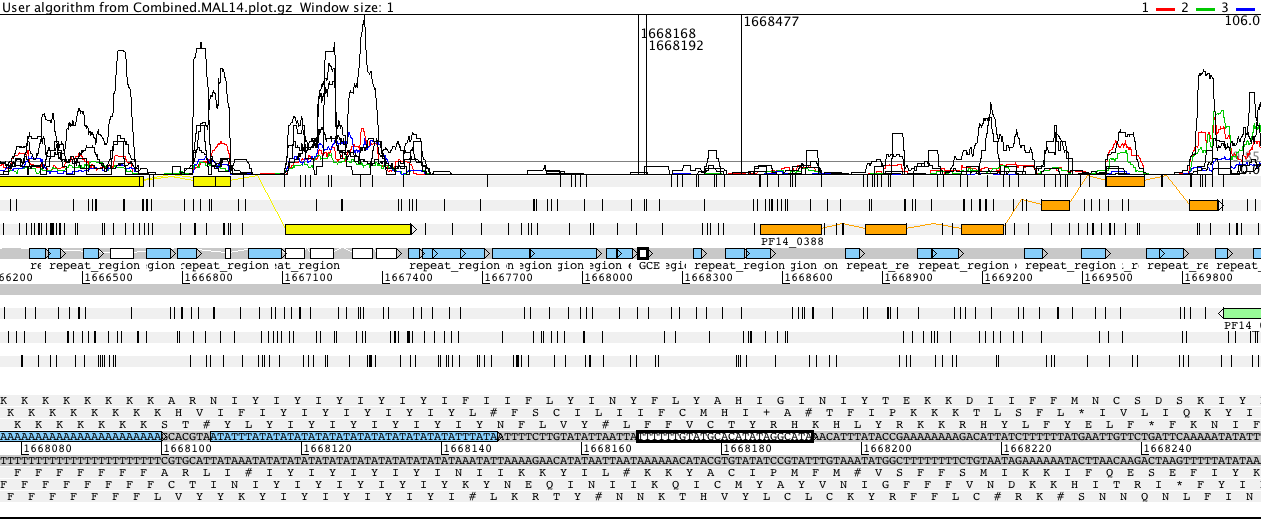


--------------------------------------------------------------------------------

Id: GCE.6.71965.72054

Pf chr: MAL14

Pf start: 1889363

Pf end: 1889452

Annotation: Pf_rna_gene Pk_rna_gene Py_intergenic

Av_blast_bit: 50.675

Av_blast_eval: 5.95e-06

PMOT: 0.297619047619048

Percent_AT: 0.662

GerpPvalue: 5.10293e-09

Gene: MAL14_48_known

Len: 90

Rnaseq_gm: 88.75

Rnaz_prob: 0.984709

CLUSTAL W(1.81) multiple sequence alignment

PlaYoe/30850-30850 C-----------------------------------------------------------

PlaCha/23129-23129 C-----------------------------------------------------------

PlaViv/45013-45102 CTTACTACATAACGTATCAATCTTTCGCCTTTTACTAAAGATTGCCGTGTAGTAGGTACA

PlaKno/49872-49961 CTTACTACATAACGTATCAATCTTTCGCCTTTTACTAAAGATTGCCGTGTAGTAGGTACA

PlaFal/40418-40507 CTTACTACATAACGAATCAATCTTTCGCCTTTTACTAAAGATTGCCGTGTAGTAAGTATG

PlaRei/40418-40507 CTTACTACATAACGAATCAATCTTTCGCCTTTTACTAAAGATTGCCGTGTAGTAAGTATG

*

PlaYoe/30850-30850 ------------------------------

PlaCha/23129-23129 ------------------------------

PlaViv/45013-45102 ATAAATACGTTATACCACAAATTTTTGTCG

PlaKno/49872-49961 CTAAATGCGTTATACCACAAATTTTTGTCG

PlaFal/40418-40507 TTAAATACAATATACCACGAATTTTTGTCG

PlaRei/40418-40507 TTAAATACAATATACCACGAATTTTTGTCG

Notes: High transcription, high RNAz SVP and annotations suggest this is a ncRNA.

Function: ncRNA

--------------------------------------------------------------------------------

Id: GCE.6.675061.675111

Pf chr: MAL14

Pf start: 2249256

Pf end: 2249306

Annotation: Pf_intergenic Pk_intergenic Py_intergenic

Av_blast_bit: 34.443

Av_blast_eval: 0.133285714285714

PMOT: 0.616071428571429

Percent_AT: 0.588

GerpPvalue: 7.31524e-20

Gene: PF14_0523 PF14_0524

Len: 51

Rnaseq_gm: 6.85

Rnaz_prob: 0

CLUSTAL W(1.81) multiple sequence alignment

PlaBer/244454-244504 TACCTGCATGATTCGAAATTTTTGAGAGACGCTAATATGACTGTTGCCTAA

PlaYoe/281887-281937 TACCTGCATGATTCGAAATTTTTGAGAGACGCTAATATGACTGTTGCCTAA

PlaCha/178870-178920 TACCTGCATGATTCGAAATTTTTGAGAGATGCTAATATGACTGTTGCCTAA

PlaViv/426862-426912 TACCTGCAGGAGGGGAAATTTTTGAGAGACGCCAAGATGACCGTTGCCTGA

PlaKno/423959-424009 TACCTGCAGGAGGGGAAATTTTTGAGAGACGCCAAGATGACCGTTGCCTGG

PlaFal/400311-400361 TACCTGCAGGATGGAAAATTTTTAAGGGAAGCTAAAATAACCGTTGCCTAA

PlaRei/400311-400361 TACCTGCAGGATGGAAAATTTTTAAGGGAAGCTAAAATAACCGTTGCCTAA

******** ** ******** ** ** ** ** ** ** *******

Notes: Now annotated as exon of PF14_0523, based on RNA-Seq data (Otto 2010).

Function: exon

--------------------------------------------------------------------------------

Id: GCE.6.1004556.1004592

Pf chr: MAL14

Pf start: 2447270

Pf end: 2447306

Annotation: Pf_intron Pk_intron Py_intron

Av_blast_bit: 36.043

Av_blast_eval: 0.125414285714286

PMOT: 0.0901420217209691

Percent_AT: 0.868

GerpPvalue: 4.81042e-06

Gene: PF14_0571

Len: 37

Rnaseq_gm: 2.18

Rnaz_prob: NA

CLUSTAL W(1.81) multiple sequence alignment

PlaBer/359696-359719 -------------AAAAAGATAAAAGAAAGGTATATT

PlaYoe/427006-427029 -------------AAAAAGGTAAAAGGAAAGTATATT

PlaCha/247516-247539 -------------AAAAAGGTAAAAGAAAGGTATATT

PlaViv/635027-635063 AAAAGAAAAAAAAAAAAAAAAAAAAAAATGGTTCACT

PlaKno/659317-659353 AAAAAAAAAAAAAAAAAAAAAAAAAAAATGGTTCACT

PlaFal/598325-598361 AAAAAGAAAAAAAAAAAAAGAAAAAAAATGATATAAT

PlaRei/598325-598361 AAAAAGAAAAAAAAAAAAAGAAAAAAAATGATATAAT

***** **** * * * *

Notes: located just before 3rd exon of PF14_0571. Low transcription. Perhaps splicing-related element? See fig.

Function: unknown


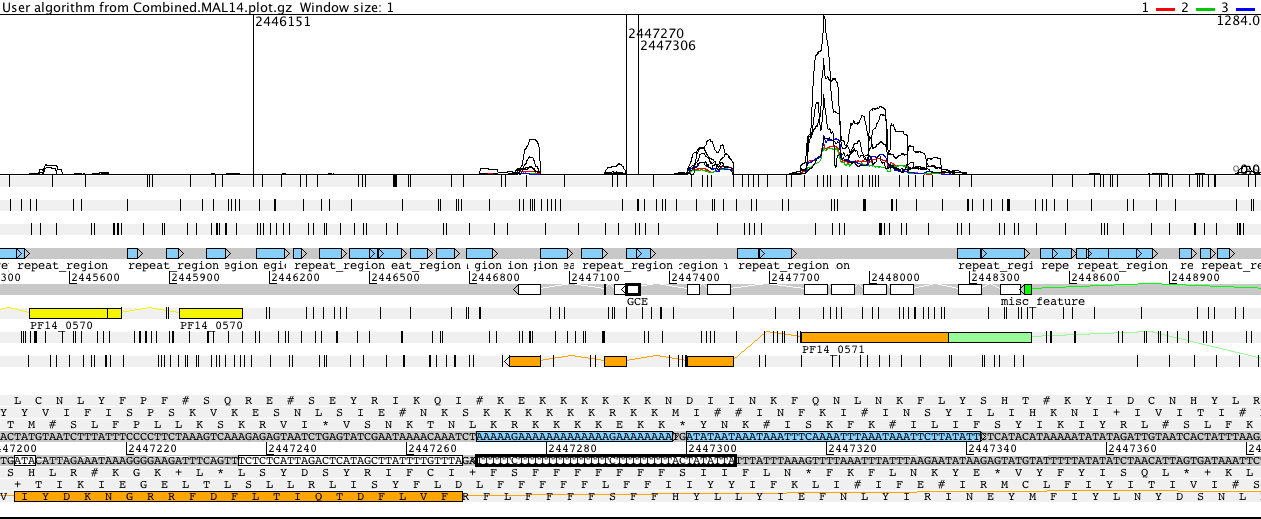


--------------------------------------------------------------------------------

Id: GCE.8.618539.618565

Pf chr: MAL14

Pf start: 2826865

Pf end: 2826891

Annotation: Pf_intergenic Pk_intergenic Py_intergenic

Av_blast_bit: 30.617

Av_blast_eval: 1.42833333333333

PMOT: 0

Percent_AT: 0.844

GerpPvalue: 1.45715e-16

Gene: PF14_0655 PF14_0656

Len: 27

Rnaseq_gm: 24.15

Rnaz_prob: NA

CLUSTAL W(1.81) multiple sequence alignment

PlaBer/160435-160456 -TTTTTTTTTTTTTTTTTTTTT----A

PlaYoe/180734-180759 -TTTTTTTTTTTTTTTTTTTTTTTTTA

PlaCha/116879-116905 TTTTTTTTTTTTTTTTTTTTTTTTTTA

PlaViv/382312-382330 -------TTTTTTTTTTTTTTTTTTT-

PlaKno/418271-418296 TTTTTTTTTTTTTTTTTTTTTTTTTT-

PlaFal/345240-345266 TTTTTTTTTTTTTTTTTTTTTTTTTTA

***************

Notes: Poly T tract. At start of highly transcribed region, that looks to be independant transcript from both genes either side. See fig.

Function: unknown


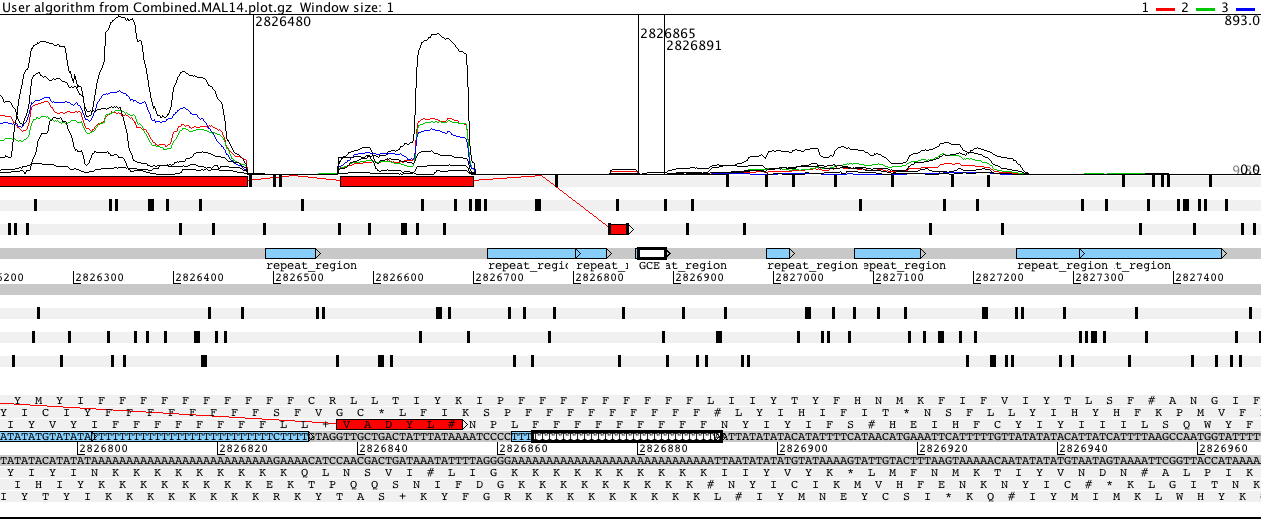


--------------------------------------------------------------------------------

Id: GCE.8.702563.702641

Pf chr: MAL14

Pf start: 2869202

Pf end: 2869280

Annotation: Pf_intergenic Pk_intergenic Py_intergenic

Av_blast_bit: 31.317

Av_blast_eval: 0.365

PMOT: 0.218566864061932

Percent_AT: 0.558

GerpPvalue: 7.87049e-09

Gene: PF14_0667 PF14_0668

Len: 79

Rnaseq_gm: 37.62

Rnaz_prob: 0

CLUSTAL W(1.81) multiple sequence alignment

PlaBer/184858-184936 TACCTTTTTTATTACGCCTGGAAAATATGCATTCATTCATATCAGGTTTTTTGTCGAAGC

PlaYoe/213749-213827 TACCTTTTTTATTACGCCTGGAAAATAAACATTCATTCATATCAGGTTTTTTGTCGAAGC

PlaCha/131049-131127 TACCTTTTTTATTACGCCTGGAAAATATACATTCATTCATATCAGGTTTTTTGTCGAAAC

PlaViv/425301-425379 TACCTTTTCTATTCCTCCTGGAGAACAAGCTCTCGCGCATATTTGGCTTCTTCTCAAAGT

PlaKno/478163-478241 TACCTTTTCTATTCCTCCTTGAGAACAAGCTCTCGCGCATATCCGGCTTCTTCTCAAAGT

PlaFal/387577-387655 TACTCTTTTGGTTTCGTCGATTAATTAAAATTTCACGCATATCAGGTTTTTTTTCAAATA

*** *** ** * * * * ** ***** ** ** ** ** **

PlaBer/184858-184936 TTGGAAAATAGCATATAGG

PlaYoe/213749-213827 TTGGAAAATAGCGTATAGG

PlaCha/131049-131127 TTGGAAAATAGCGTATAGG

PlaViv/425301-425379 TGGGAAAATAACGTATCAC

PlaKno/478163-478241 TCGGAAAATAACGTATCAC

PlaFal/387577-387655 CTGGTATATACTGTATATA

** * *** ***

Notes: Now annotated as exon of PF14_667a:1:pep, from RNA-Seq data (Otto 2010).

Function: exon

--------------------------------------------------------------------------------

Id: GCE.8.824688.824800

Pf chr: MAL14

Pf start: 2942076

Pf end: 2942182

Annotation: Pf_intergenic Pk_intergenic Py_intergenic

Av_blast_bit: 39.371

Av_blast_eval: 0.442858142857143

PMOT: 0.547327029007047

Percent_AT: 0.664

GerpPvalue: 9.51347e-17

Gene: MAL14_69_candidate_ncRNA PF14_0689

Len: 113

Rnaseq_gm: 116.96

Rnaz_prob: 0

CLUSTAL W(1.81) multiple sequence alignment

PlaBer/241698-241809 ATATTTACCTTAAATTAATAAGAATAGAAATAAATGACAAAATAACTAACGGGAGTAAGG

PlaYoe/259236-259347 ATATTTACCTTAAATTAATAAGAATAGAAATAAATGACAAAATGACTAACGGGAGTAAGG

PlaCha/173821-173933 ATATTTACCTTAAATTAATAAGAATAGAAATAAATGACAAAATAACTAACGGGAGTAAGG

PlaViv/500175-500287 CCCCTTACCTCAAATTGATAATTATCGAAATGAAGGACAAAATGACCAGCGGGAGCAGCG

PlaKno/552026-552138 CCCTTTACCTCAAATTGATAATTATTGAAATGAAGGATAAAATGACCAGTGGAAGTAGTG

PlaFal/460451-460557 -TATTTACCTTAAATTAATAATTATAGATATTAATGATAATATGACTAGAGGTAACAGAG

PlaRei/460451-460557 -TATTTATCCTAAATTAATAATTATAGATATTAATGATAATATGACTAGAGGTAACAGAG

*** * ***** **** ** ** ** ** ** ** ** ** * ** * * *

PlaBer/241698-241809 CGTACCCAAGCATGCTTATTGTACAATATAGATCTAAAGTTGCGTTCTG-ATT

PlaYoe/259236-259347 CATACCCAAGCATGCTTATTGTGCAATATAAATCTAAAGTTGCATTCTG-ATT

PlaCha/173821-173933 CATACCCGAGCATGCTTATTGTGCAATATAGATCTAAAGTTGCATTCTGAATT

PlaViv/500175-500287 CGTAGCCCAGCATGCTTATCGTGCGGTACAGGTCCAGCGTCAGGCTCTGAAGT

PlaKno/552026-552138 CATAGCCAAGCATGCTTATCGTGCGGTACAGGTCCAGCGTCAGATTCTGAAGT

PlaFal/460451-460557 CATAACCGAGCATACTAATGGTTCGATATAAATCCACAGTTGAATTCT-----

PlaRei/460451-460557 CATAACCGAGCATACTAATGGTTCGATATAAATCCACAGTTGAATTCT-----

* ** ** ***** ** ** ** * ** * ** * ** ***

Notes: Recently annotated as exon of PF14_0689. High expression, PMOT and blast support this.

Function: exon

--------------------------------------------------------------------------------

Id: GCE.9.73911.73946

Pf chr: MAL14

Pf start: 3066946

Pf end: 3066981

Annotation: Pf_intergenic Pk_intergenic Py_intergenic

Av_blast_bit: 32.171

Av_blast_eval: 1.58157142857143

PMOT: 0.313795518207283

Percent_AT: 0.906

GerpPvalue: 7.98182e-05

Gene: PF14_0715 PF14_0716

Len: 36

Rnaseq_gm: 34.05

Rnaz_prob: NA

CLUSTAL W(1.81) multiple sequence alignment

PlaBer/21220-21254 TTTTTTATTA-TTTAATTATTGTTTTTCACATTTTT

PlaYoe/26305-26339 TTTTTTATTA-TTTAATTATTGTTTTTCACATTTTT

PlaCha/12484-12519 TTTTTTATTATTTTAATTATTGTTTTTCACATTTTT

PlaViv/42268-42292 TTTTT-GTGCCTTT-------GTTTTTT---TTTTT

PlaKno/42704-42736 TTTTTTGTGCCTTTTTTTTTTTTTTTTT---TTTTT

PlaFal/56426-56461 TTTTTTCTTTCTTTATTTATTTTTTATTATATATTT

PlaRei/56426-56461 TTTTTTCTTTCTTTATTTATTTTTTATTATATATTT

***** * *** *** * * ***

Notes: At start of highly transcribed region with many stop codons. Could be untranslated exon of PF14_0716. See fig.

Function: unknown, UTR?


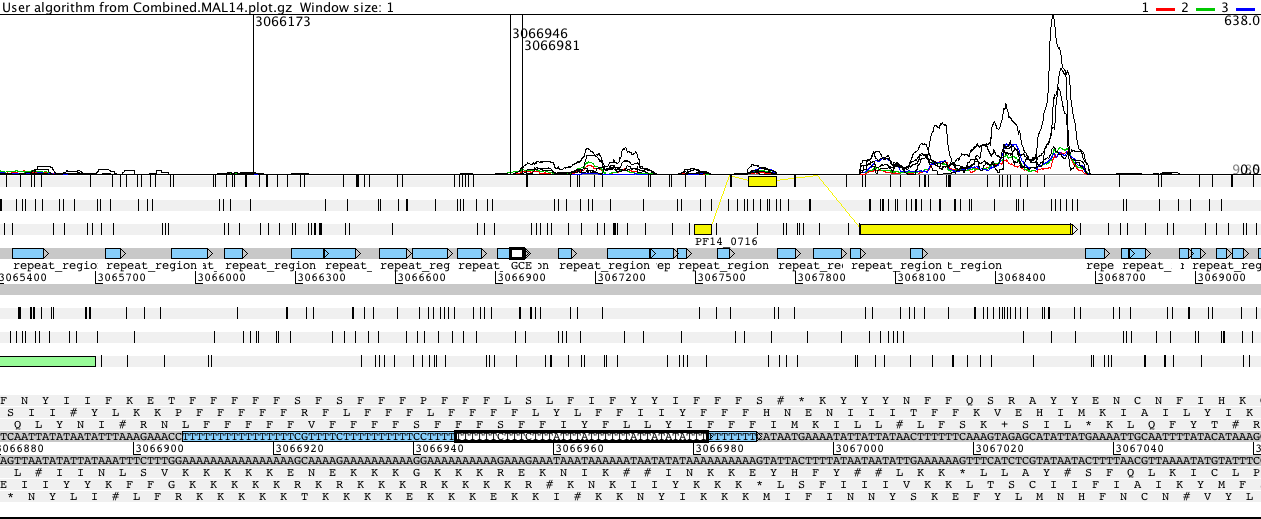

Supplement: Text S1 — (0.41 MB DOC) [file pgen.1001099.s014.doc]
